# Cost analysis based on bioreactor cultivation conditions: Production of a soluble recombinant protein using *Escherichia coli* BL21(DE3)

**DOI:** 10.1016/j.btre.2020.e00441

**Published:** 2020-02-22

**Authors:** Valdemir M. Cardoso, Gilson Campani, Maurício P. Santos, Gabriel G. Silva, Manuella C. Pires, Viviane M. Gonçalves, Roberto de C. Giordano, Cíntia R. Sargo, Antônio C.L. Horta, Teresa C. Zangirolami

**Affiliations:** a Graduate Program of Chemical Engineering (PPGEQ), Federal University of São Carlos (UFSCar), Rodovia Washington Luís, km 235, 13565-905, São Carlos, SP, Brazil; b Department of Engineering, Federal University of Lavras, 37200-000, Lavras, MG, Brazil; c Laboratory of Vaccine Development, Butantan Institute, Av. Vital Brasil 1500, 05508-900, São Paulo, SP, Brazil; d Brazilian Biorenewables National Laboratory (LNBR), Brazilian Center for Research in Energy and Materials (CNPEM), 13083-970, Campinas, SP, Brazil

**Keywords:** Batch culture, Energy consumption, Process economic analysis, Stirred-Tank reactor, Bioprocess engineering

## Abstract

The impact of cultivation strategy on the cost of recombinant protein production is crucial for defining cost-effective bioreactor operation conditions. This paper presents a methodology to estimate and compare cost impacts related to utilities as well as medium composition, using simple design equations and accessible data. Data from batch bioreactor cultures were used as case study involving the production of pneumococcal surface protein A, a soluble recombinant protein, employing *E. coli* BL21(DE3). Cultivation strategies and corresponding process costs covered a wide range of operational conditions, including different media, inducers, and temperatures. The core expenses were related to the medium and cooling. When the price of peptone was above the threshold value of US$ 30/kg, defined medium became the best choice. IPTG and temperatures around 32 °C led to shorter cultures and lower PspA4Pro production costs. The procedure offers a simple, accessible theoretical tool to identify cost-effective production strategies using bioreactors.

## Highlights

• Methodology of cost analysis based on process conditions was presented and detailed.
• The core expenses were related to medium and cooling.
• Energy costs were mainly related to the extent of the cultivation.
• Induction with IPTG and temperatures around 32 °C presented lower production costs.
• The price of peptones had a strong influence on the cost of the complex media.

## Graphical abstract

**Figure:**
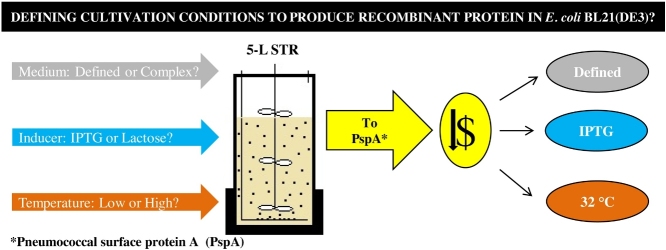

## Nomenclature

ANS: Animal nitrogen source
C_k_^Comp^: Concentration of component k in the medium
CD: Circular dichroism
CM: Complex medium
Cx: Biomass concentration, g_DCW_/L
D_i_: Impeller diameter, 0.078 m
DCW: Dry cell weight
DM: Defined medium
D_R_: Reactor diameter, 0.160 m
DOT: Dissolved oxygen tension
E_C_: Total energy consumption of compressor, kWh
E_S_: Total energy consumption of stirring, kWh
E_M_: Total metabolic energy generation, kWh
Exp #: Experiment number
f_C_: Correction factor
g: Gravitational acceleration, m/s^2^
H_i_: Impeller height, 0.267 m
H_R_: Reactor height, m
IPTG: Isopropyl-β-d-thiogalactopyranoside
K: Constant of proportionality, 50 kJ/mmol O_2_
LAC: Lactose
MC: Medium cost
N: Stirring speed, rpm or s^−1^
N_p_: Power number, 5.2 (Rushton impeller in turbulent regime)
∑n_AIR_: Total air amount during the experiment, mol
n_C_^max^: Maximum air amount into compressor, mol
n_C_^min^: Minimum air amount into compressor, mol
OD: Optical density
OUR: Oxygen uptake rate, mmol O_2_/h
p: Pressure of entering gas, atm
P: Power of ungassed stirring, kW
p_1_: Inlet absolute pressure of compressor, Pa
p_2_: Outlet absolute pressure of compressor, Pa
P_B_: Power consumption of thermostatic bath, 0.710 kW
P_C_: Power consumption of compressor, kW
P_P_: PspA4Pro production, g/L
P_S_: Power consumption of stirring, kW
PspA4Pro: Untagged pneumococcal surface Protein A from clade 4
Q_1_: Total entering volumetric gas flow, L/min
Q_AIR_: Air volumetric flow rate
Q_G_: Total gas volumetric flow rate, m^3^/s
Q_Heat_: Metabolic heat release rate, kJ/h
Q_O2_: Oxygen volumetric flow rate
R: Gas constant, 0.082 atm.L/mol.K
$_i_: Cost of i, where i is gases, cooling, stirring, or medium, US$/(g_DCW_ or g_PspA_)
$_k_^Comp^: Cost of component k in the medium
STR: Stirred tank reactor
T: Culture temperature
T_1_: Temperature of entering gas streams, °C
t_f_: Cultivation time
VNS: Vegetal nitrogen source
V_O2_: Total oxygen consumption volume, m^3^
V_R_: Bioreactor culture volume, L

**Greek letters**
ρ_b_: Broth density, kg/m^3^
η_B_: Efficiency number of thermostatic bath, 0.7
η_C_: Efficiency number of compressor, 0.70
η_S_: Energy efficiency, 0.65
γ: Isoentropic exponent, 1.4 for oxygen

**Superscripts**
max: Related to a maximum value
min: Related to a minimum value
‘^’: Indicator that geometries are from the STR used in the experiments

**Subscript**
f: Values related to tf

## 1 Introduction

Therapeutic recombinant proteins are used for treating a variety of diseases [1,2]. Recombinant proteins are also used as antigens of subunit vaccines, among which recombinant antigens of *Neisseria meningitidis* and cholera toxin B subunit are produced in *E. coli* [3]. There is also an enormous number of subunit vaccine candidates for different pathogens, such as dengue virus [4], *Leptospira* [5], *Leishmania* [6]*, Streptococcus pneumoniae* [7], *Mycobacterium tuberculosis* [8], and *Helicobacter pylori* [9], among others, several of them produced as recombinant proteins in *E. coli*.

Almost a third of therapeutic recombinant products have been produced using *E. coli* [2,10,11], demonstrating the importance of this microorganism as an expression host. *E. coli* not only has a well-characterized genome, but is also easily genetically manipulated [1], which has enabled the expression of several different recombinant proteins [12,13] and metabolites [14,15]. Furthermore, *E. coli* presents rapid growth and high biomass formation yield, as well as low production costs [16,17], favoring its industrial scale use, compared to other possible hosts [18].

The production of recombinant proteins using *E. coli* has been widely reported. A huge number of studies have focused only on cloning and heterologous protein production at the shake flask scale [[19], [20], [21]]. At the bioreactor scale, most works have focused on cultivation strategies to achieve high cell and protein concentrations [22,23]. There have also been a few studies addressing the impact of different cultivation techniques on process economics [[24], [25], [26], [27], [28], [29], [30], [31]]. Defined or complex media can be used during cultivations [23]. On one hand, a defined medium has been suggested as the best choice for production of therapeutic recombinant proteins [32], since its known composition enables the concentration of individual components to be followed, resulting in better bioreactor control. On the other hand, complex media can boost product formation [22] and provide higher growth rates [33], compared to defined media. For these reasons, complex media are widely used in industry [18], including for the production of therapeutics. However, in this case, complex medium formulations require prion-free certified animal-based nitrogen sources (such as tryptones), or certified nitrogen sources derived from plants and microbes (such as yeast extract and soybean peptones). Hence, the choice between complex and defined media is controversial and its economic impact goes beyond the corresponding costs of the medium components. In fact, together with temperature, culture medium and inducer will influence the duration of cultivation, protein synthesis, and biomass formation. So, shedding light on the impact of medium formulation on overall bioreactor economics would help in deciding which medium to use for recombinant protein production in *E. coli*.

Since the aim of industrial fermentation is to obtain high yields using inexpensive raw materials, with low capital costs [34], cost evaluation may provide a convenient guide for selection of a cultivation strategy [32]. Different approaches can be used for this purpose. It has been reported that the replacement of batch processes by continuous ones may be a natural option for decreasing costs and raising productivity [30]. Other studies have focused on the simulation of new configurations of biopharmaceutical plants and estimates of payoff times [25,31]. Comparison has been made of alternative medium formulations for growth of *E. coli* as a host organism [26,28]. Although Fong and Wood [28] considered the use of isopropyl-β-d-thiogalactopyranoside (IPTG) and cheaper nitrogen and carbon sources, the cost impacts of the inducer and alternative nitrogen and carbon sources were only evaluated qualitatively. Ferreira et al. [27] estimated the quantitative economic impacts of these materials by simulation, although their influences on process dynamics were not demonstrated experimentally. The studies undertaken by Campani et al. [24] and Knoll et al. [29] focused on bioreactor utilities, with theoretical analyses of costs related to mixing, compression, and the supply of pure oxygen. Until now, for recombinant protein production in bioreactors, the combined cost implications of different media, inducers, nitrogen sources, and cultivation temperatures, together with the associated utilities expenses, have not been considered or published in a single paper, in order to facilitate the incorporation of such a methodology in the selection of bioreactor cultivation conditions at laboratory or industrial scales.

Although different types of recombinant proteins may present particular process sets that enable achievement of better production results [35,36], efforts are needed to understand how bioreactor operational conditions impact the overall economic performance of heterologous protein production processes. The untagged fragment of pneumococcal surface protein A (PspA4Pro) is a potential candidate for a serotype-independent vaccine against *Streptococcus pneumoniae* [37] and its production has been studied using recombinant *E. coli* cultivations carried out under different strategies [24,38,39]. PspA is a single chain α-helix-rich protein that binds to human lactoferrin [40] and to the C3 molecule of the serum complement [41], contributing to *S. pneumoniae* evading the host immune system. Recombinant fragments of PspA, which contain the N-terminal α-helix domain, the proline-rich region, and no disulfide bonds, are highly soluble [[42], [43], [44]] and present a coiled-coil structure [45,46]. This structure confers stability and thermoresistant characteristics to the molecule [37].

Thus, this work presents a procedure for implementing an overall cost analysis, considering the integrated effects of different media, complex nitrogen sources, temperatures, and inducers on the associated costs (for cooling, stirring, air compression, and supply of pure oxygen), based on bioreactor cultivation conditions and theoretical design equations. As a case study, the proposed calculation procedure was applied to carry out an overall economic analysis of process performance for biomass and PspA4Pro protein production during batch stirred-tank bioreactor cultures. The procedure could be easily extended to economic assessments for other bioproducts and bioreactor scales.

## 2 Materials and methods

### 2.1 Experimental data

All experiments were carried out with *E. coli* BL21(DE3) (Invitrogen, Carlsbad, CA, USA) harboring the plasmid pET37b(+)/*psp*A4Pro, which carries the gene encoding an untagged fragment of the pneumococcal surface protein A (PspA) gene from family 2, clade 4, named PspA4Pro [44]. The expression of the PspA4Pro gene is controlled by the *lacUV5* and *T7lac* promoters, with the soluble recombinant protein being accumulated intracellularly after the inducer (IPTG or lactose) is added [37,38]. The recombinant *E. coli* strain was kindly provided by Dr. Eliane Miyaji from the Laboratory of Molecular Biology, Butantan Institute (São Paulo, Brazil).

Although there are other factors that can play a role in recombinant protein production using *E. coli* bioreactor cultures, such as duration of induction, timing of induction, inducer concentration, and dissolved oxygen tension, among others [36,47], in this work, temperature, type of inducer, and medium composition were chosen to evaluate the application of the proposed methodology to the economics of PspA4Pro production and to compare cultivation strategies. Experimental data for the cost analysis were obtained from nine bioreactor cultures carried out under different cultivation and induction conditions, using complex and defined media, different complex nitrogen sources, lactose and IPTG (in defined medium) as inducers, and cultivation temperatures from 27 to 37 °C (Table 1).

**Table 1:** Main *E. coli* BL21(DE3) pET37b(+)/*psp*A4Pro cultivation conditions for batch bioreactor Experiments #1 to #8, cultivation time (t_f_) to reach the highest PspA4Pro production (P_P_), and the corresponding biomass concentration (C_X_). Data for Experiments #2, #5, #7 (I and II), and #8 are available as Supplementary Material.

Exp #	Medium/T/Inducer	t_f_	C_X_	P_P_	Reference
		h	g_DCW_/L	g_PspA4Pro_/L	
1	DM/27 °C/IPTG	25.5	32.8 ± 1.8	3.1 ± 0.5	[38]
2	DM/27 °C/LAC	27.5	34.5 ± 0.6	2.6 ± 0.3	This work
3	DM/32 °C/IPTG	16.3	31.3 ± 1.6	3.8 ± 0.4	[38]
4	DM/37 °C/IPTG	14.6	27.0 ± 1.0	3.4 ± 0.3	[38]
5	DM/37 °C/LAC	19.5	20.4 ± 0.3	1.3 ± 0.0	This work
6	CM-ANS/31 °C/LAC	16.4	38.0 ± 0.7	5.4 ± 0.3	This work
7.I	CM-VNS/31 °C/LAC	16.6	38.3 ± 0.7	5.6 ± 0.3	[37]
7.II	CM-VNS/31 °C/LAC*^a^*	16.0	39.2 ± 0.7	6.0 ± 0.3	This work
8	CM-VNS/31 °C/LAC*^b^*	15.0	31.8 ± 2.3	4.7 ± 0.7	This work

CM-ANS: Complex medium with animal nitrogen source; DCW: Dry cell weight; DM: Defined medium; Exp #: Experiment number; LAC: lactose; PspA4Pro: Pneumococcal surface Protein A from clade 4; T: Culture temperature; CM-VNS: Complex medium with vegetal nitrogen source. a: Replication of experiment #7.I; b: Alternative vegetal nitrogen source.

The first stage of the study used a factorial design approach to evaluate the cost effects of temperature and inducers in PspA4Pro production using batch bioreactor cultivations employing moderate biomass concentrations. Experiments #1 to #5 were batches performed with HDF medium [48], at 27, 32, and 37 °C, using glycerol as sole carbon source and isopropyl-β-d-thiogalactopyranoside or lactose as inducer. After identifying the most promising temperature, additional experiments using complex medium at this temperature were carried out in order to enable comparison of the effects of defined or complex cultivation media on the cost of PspA4Pro production in the bioreactor. In the second stage, investigation was made of the performance achieved using different complex nitrogen sources obtained from the hydrolysis of animal and vegetal proteins. Experiments #6 to #8 comprised a set of bioreactor batches carried out with modified ZYM-5052 auto-induction complex medium [49]. Tryptone (enzymatic hydrolysate of casein) was the animal nitrogen source used in Experiment #6, while soy peptone (enzymatic papain digest of soybean protein) and a homemade soybean protein supplement extract were used in Experiments #7 (a and b, as duplicates) and #8, as the vegetal nitrogen sources. The latter experiment was performed using the extract of an inexpensive soybean protein source, in order to determine the influence of feedstock quality on recombinant protein production and its related costs. Lactose was used as inducer in all experiments carried out with complex media under the auto-induction strategy.

Details about the procedure and the results of each experiment can be found in the references provided in Table 1, or in the Supplementary Material. The compositions of all the media employed in the experiments are presented in Table 2.

**Table 2:** Concentrations and costs of each medium component used in the bioreactor cultures.

Component	Purchase Cost (US$/kg)		Concentrations for each experiment		
		Units	1, 3, and 4	2 and 5	6, 7, and 8
Glucose*^a^*	0.7	g/L			10.0
Glycerol*^a^*	1.0	g/L	60.0	60.0	60.0
Yeast extract*^a^*	1.7	g/L			5.0
MgSO_4_.7H_2_O*^a^*	0.3	g/L	0.4	0.4	0.5
Na_2_HPO_4_*^a^*	2.0	g/L			9.0
KH_2_PO_4_*^b^*	1.2	g/L	17.7	17.7	3.4
NH_4_Cl*^a^*	0.2	g/L			2.7
(NH_4_)_2_HPO_4_*^a^*	0.4	g/L	5.3	5.3	
Citric acid*^a^*	1.2	g/L	2.3	2.3	
Na_2_SO_4_*^a^*	0.1	g/L			0.7
Kanamycin*^b^*	1.0	mg/L	100.0	100.0	100.0
Ferric citrate*^b^*	6.0	mg/L	133.3	133.3	100.8
CoCl_2_.6H_2_O*^b^*	12.7	mg/L	3.3	3.3	2.5
MnCl_2_.4H_2_O*^b^*	1.7	mg/L	20.0	20.0	15.0
CuCl_2_.2H_2_O*^a^*	4.2	mg/L	2.0	2.0	1.5
H_3_BO_3_*^a^*	0.6	mg/L	4.0	4.0	3.0
NaMoO_4_.2H_2_O*^a^*	9.9	mg/L	2.8	2.8	2.1
Zn(CH_3_CHOOH).H_2_O*^b^*	1.7	mg/L	33.8	33.8	33.8
EDTA*^a^*	1.9	mg/L	18.8	18.8	14.1
Thiamine*^a^*	36.1	mg/L	45.0	45.0	45.0
Polypropylene glycol*^a^*	3.6	g/L	0.3	0.3	0.3
Peptone*^a^*	6.1	g/L			10.0
Soy supplement*^c^*	12.2	g/L			10.0
Lactose*^a^*	2.0	g/L		20.0	20.0
IPTG*^b^*	601	mmol/L	1.0		
**Total medium expenses**		**US$/L**	**0.24**	**0.15**	**0.21*d*/0.27*e***

Sources of raw material costs: a: COSTDRIVERS; b: Molbase; c: Soy supplement supplier. d: Peptone (Experiments #6 and #7); e: Soy Supplement (Experiment #8).

#### 2.1.1 Bioreactor cultivation

The following experimental procedure was employed in all experiments. A pre-inoculum was prepared from a single bacterial colony transferred from an LB-Miller-agar-kanamycin plate to 50 mL of the desired liquid medium plus kanamycin (50 mg/L), in a 0.5 L Erlenmeyer flask. The flask was kept overnight at 37 °C, with agitation at 250 rpm, in a controlled temperature incubator-shaker (New Brunswick), until reaching optical density (OD) of 2.0 (at 600 nm). Under the same conditions, an inoculum was prepared from a volume of the pre-inoculum necessary to reach OD of 0.1 in 300 mL of the fresh medium under investigation, distributed equally in three 0.5 L Erlenmeyer flasks. When OD of 2.0 was reached, the inoculum was transferred to the bioreactor containing approximately 4 L of the medium under investigation. A 5 L stirred-tank reactor (STR), described in detail elsewhere [50], was used for all the cultures listed in Table 1. The bioreactor was controlled and monitored using SuperSys_HCDC software and the on-line data were logged using an analog-to-digital converter (Model cFP2020, National Instruments). The pH was automatically controlled at 6.7 (growth phase) or 6.9 (induction phase), using solutions of NH_4_OH (15 % v/v) and HCl (9% v/v). Dissolved oxygen tension (DOT) was measured with an amperometric probe (Model InPro 6830, Mettler Toledo) and was maintained at 30 % of saturation by adjusting the stirrer speed (from 200 to 900 rpm) and the two mass flow controllers (GFC, Aalborg) governing the air and oxygen flow rates. Air and oxygen were supplied to the bioreactor from a compressor (Model MAW-60/425, GMEG) and a high-pressure gas cylinder (White Martins), respectively, reaching a maximum total gas flow rate of 4–6 std L/min (at 21.1 °C and 1 atm). The culture temperature was measured with a thermocouple (Pt-100, Exacta) and maintained at the desired set-point by continual indirect heat transfer using water from a thermostatic bath (0.7 kW, Solab) passed through the bioreactor jacket.

#### 2.1.2 Analyses of culture samples

The bioreactor culture biomass and PspA4Pro concentrations used in the cost estimation are also shown in Table 1. The biomass concentration (C_X_) was monitored by optical density (OD) measurements at 600 nm and by the dry cell weight method [51,52]. Plasmid loss during the cultivations was evaluated using diluted samples (1:10^6^ or 1:10^7^) of culture broth spread onto LB-Miller-agar plates [24].

PspA4Pro production (P_P_) was assessed by band densitometry after cell disruption (by sonication) and clarification [24]. The total soluble protein concentration for the known cell concentration of the disrupted sample was determined by the Bradford method [53]. The proteins present in the clarified sample were identified by 12 % SDS-PAGE [54]. The soluble recombinant protein fraction in the clarified sample was estimated using ImageJ software to analyze images of the gel stained with Coomassie Blue R [55], together with determination of the total soluble protein concentration as described by Campani et al. [24]. In order to confirm the absence of insoluble PspA4Pro as inclusion bodies, pellets obtained after sonication were resuspended in 2 mL of buffer (20 mM TRIS; 250 mM NaCl; pH 8.0), mixed with Laemmli buffer (1:2) [54], boiled at 100 °C for 15 min, and applied to 12 % SDS-PAGE plates.

#### 2.1.3 PspA4Pro secondary structure and lactoferrin binding assay

Additional methods to characterize the quality of the proteins obtained by different cultivation strategies were applied to samples from Experiments #2, #3, #4, #7, and #8. To this end, cell pellets harvested at the end of bioreactor cultures #2, #3, #4, #7, and #8 were frozen (at −80 °C), followed by processing according to the purification procedure described previously [37]. The pure recombinant PspA4Pro obtained from the soluble fraction of each biomass was analyzed by circular dichroism (CD) to confirm the PspA4Pro secondary structure, according to the method described previously [37].

The PspA4Pro biological activity was evaluated by a lactoferrin binding assay using 96-well flat-bottom plates (MaxiSorp, Nunc). The plates were coated with 2 μg/well of human lactoferrin (L1294, Sigma), incubated overnight at 4 °C, washed 3 times with phosphate buffered saline + 0.05 % Tween 20 PBS-T, and blocked with 5% skimmed milk. The plates were then washed with PBS-T and incubated at 37 °C for 2 h, with serial dilutions, in 5% skimmed milk, of PspA4Pro purified from each experiment. Blank wells received only 5% skimmed milk, while the positive control was a PspA4Pro standard. After washing with PBS-T, anti-PspA4Pro rabbit serum 1:5000 in 5% skimmed milk was added and the plates were incubated at 37 °C for 1 h, washed with PBS-T, and incubated with anti-rabbit IgG conjugated to peroxidase A0545, Sigma, 1:5000 in 5% skimmed milk. The color was developed for 15 min, at room temperature, in the presence of o-phenylenediamine and hydrogen peroxide, and the reaction was stopped with 4 M H_2_SO_4_. The absorbance measured at 492 nm was plotted against the PspA4Pro concentration, with the slope being used as a parameter for the lactoferrin binding property of PspA4Pro. The anti-PspA4Pro antibody was obtained in rabbit immunized with 3 doses, at two week intervals, of 200 μg of previously purified PspA4Pro [37] absorbed in 5 mg of aluminum hydroxide. The immunization and bleeding protocols followed the rules issued by the National Council for Control of Animal Experimentation CONCEA and were approved by the Ethics Committee on Animal Use of the Butantan Institute CEUAIB nº 7,755,300,718.

### 2.2 Direct cost estimation

In order to assess the influence of different cultivation conditions on the process economics, the direct costs related to cell and recombinant protein production were evaluated by taking into account the costs of raw materials (pure oxygen, inducers, and medium components) and utilities (based on energy consumption for cooling, stirring, and air compression). Effective theoretical methodologies to estimate these costs were obtained from several literature sources and were compiled in a spreadsheet to enable simple and accessible implementation of the evaluations. The proposed procedure could be applied to estimate costs not only for recombinant proteins, but also for any bioproduct, provided that a cultivation monitoring system is available for data acquisition, as described in the bioreactor cultivation section. Furthermore, this cost analysis methodology could be applied to compare bioreactor operational conditions, regardless of the bioreactor scale.

A flowchart summarizing the main steps of the cost analysis implementation is provided in the Supplementary Material (Figure S1). In addition, a simple version of the spreadsheet is available as a workstation for fast cost evaluations of bioreactor cultures and for teaching purposes. The component values of this workstation may be updated by users, in order to make the results of each analysis more realistic, depending on the criteria adopted. Despite contributing to the total cost of a fermentation process, costs related to sterilization, equipment depreciation, and employees were not considered in this work, because they remained almost the same, regardless of the cultivation conditions evaluated in the experiments.

As described next, the direct cost was estimated using Eq.s 1 to 11 and data from bench-scale bioreactor cultures (Section 2.1.1). For each production strategy, the direct cost was evaluated at the cultivation time corresponding to the maximum recombinant protein concentration (Table 1). Direct cost values were presented as ratios relative to Experiment #3, which corresponded to the lowest direct cost for PspA4Pro production using defined medium, in the present experiments.

#### 2.2.1 Medium costs

The medium costs were estimated using Eq. 1, considering the initial amounts of the components added to formulate the medium, together with their market prices (Table 2). Although all the experiments were performed with laboratory-grade products purchased in small quantities, the costs of most of the medium components were estimated using information obtained from business intelligence and e-commerce platforms [56,57], which provided a better representation of market prices. Together with the concept of “direct cost ratio”, the standardization of the price survey was important to ensure the data consistency required to compare the cultivation conditions investigated. An exception was the soy protein supplement used in Experiment #8, for which the sale price was used directly. The prices of casein and soybean-based peptone, which are indicated in Table 2 as animal-based (ANS) and vegetal (VNS) nitrogen sources, respectively, were also retrieved from the platforms, where they were identified generically as “peptone”. The market price of peptone varied from 6 to 300 US$/kg, depending on its nutritional value as a microbial culture supplement, solubility, and quality certification, as well as the amount purchased and the supplier. Oxoid LP0042 Tryptone was used in Experiment #6, BD BBL Phytone was used in Experiments #7.a and #7.b, and Growth Supplements soy protein supplement was used in Experiment #8. This nitrogen source required additional preparation steps for its solubilization, before being added to the medium (details are provided in the Supplementary Material).

(1) $${\$}_{MEDIUM}=\;\frac{1}{V_{R}(C_{X\;}or\;P_{P})}\sum_{1}^{n}(C_{K}^{Comp}{\$}_{K}^{Comp})$$

$_MEDIUM_: Cost of medium, US$/(g_DCW_ or g_PspA4Pro_);

C_X_: Biomass concentration, g_DCW_/L;

C_k_^Comp^: Concentration of component k in the medium (Table 2);

P_P_: PspA4Pro production, g/L;

$_k_^Comp^: Cost of component k in the medium (Table 2) converted according to C_k_^Comp^.

#### 2.2.2 Cost of air and oxygen supplies (gases)

In order to maintain DOT at 30 % of saturation, a sequential control strategy was applied using the Supersys_HCDC software, comprising stepwise increases in stirring speed and air volumetric flow rate (Q_AIR_), up to their upper limits, followed by the gradual enrichment of air with pure oxygen. This enrichment was accomplished by manipulation of the oxygen (Q_O2_) and air volumetric flow rates, so that the total inlet flow rate of the gas supplied to the bioreactor was kept at 4–6 std L/min [58]. The Q_O2_ and Q_AIR_ values were recorded by Supersys_HCDC, enabling determination of the total volume of oxygen supplied (V_O2_ = ƩQ_O2_.Δt) and the total energy consumption of the compressor (E_C_ = ƩP_C_.Δt), with these two values being used to determine the gases costs. The overall cost of supplying air and oxygen is given by Eq. 2, where the market prices for oxygen and electricity were US$ 0.52/std m^3^ and US$ 0.126/kWh, respectively [56]. The compressor power consumption (P_C_) was estimated theoretically using Eq. 3. This approach was analogous to the procedure of Knoll et al. [29], considering an ideal single-stage compressor, with electrical and mechanical compression losses taken into account using the efficiency number (η_C_ = 0.7) [24].

(2) $${\$}_{GASES}=\;\frac{1}{V_{R}\left(C_{X\;}or\;P_{P}\right)}(0.52V_{02}+0.126E_{C})$$

$_GASES_: Cost of gases, US$/(g_DCW_ or g_PspA4Pro_); V_R_: Bioreactor culture volume, L; V_O2_: Total oxygen consumption volume, m^3^; E_C_: Total energy consumption of compressor, kWh.

(3) $$P_{C}=\;\frac{p_{1}Q_{AIR}}{\eta_{C}}\frac{\gamma }{\left(\gamma -1\right)}\left\{{\left(\frac{p_{2}}{p_{1}}\right)}^{\left[\frac{\left(\gamma -1\right)}{\gamma }\right]}-1\right\}$$

P_C_: Power consumption of compressor, kW; Q_AIR_: Air volumetric flow rate, L/min; p_1_: Inlet absolute pressure of compressor, Pa; p_2_: Outlet absolute pressure of compressor, Pa; η_C_: Efficiency number of compressor [24]; γ: Isoentropic exponent, 1.4 for oxygen [24].

#### 2.2.3 Cost of stirring

The cost of stirring was determined using Eq. 4. The total energy consumed for stirring (E_S_) was calculated by integrating over time the theoretical values of gassed stirring power consumption, P_S_ (E_S_ = ƩP_S_.Δt). These values were correlated to the power for ungassed stirring (P), using Eqs. 5 or 6 [59]. The energy efficiency (η_S_) was assumed to be 0.65 [24] and the impellers were considered to be operated at maximum velocity (900 rpm), since this condition would demand the highest energy consumption, in order to better represent an overdesigned engine. Considering three impellers immersed in the liquid, the P values were determined using Eqs. 7 and 8, according to the Rushton method combined with the Michel and Miller correlation [60] and the correction factor (f_C_) used by Campani et al. [24], assuming standard STR geometries of diameter (D_R_/D_i_) and height (H_R_/D_i_) equal to 3. For Eqs. 5 and 6, the total volumetric gas flow rate values were retrieved from the dataset automatically acquired by the SuperSys_HCDC software.

(4) $${\$}_{S}=\;0.126\frac{1}{V_{R}\left(C_{X\;}or\;P_{P}\right)}E_{S}$$

$_S_: Cost of stirring, US$/(g_DCW_ or g_PspA4Pro_);

E_S_: Energy consumption of stirring, kWh.

(5) $${\;P}_{S}=\;\frac{P}{\eta_{S}}\left(1-12.2\frac{Q_{G}}{ND_{i}^{3}}\right),\;valid\;for\;\frac{Q_{G}}{ND_{i}^{3}}<0.037$$

(6) $${\;P}_{S}=\;\frac{P}{\eta_{S}}\left(0.62-1.85\frac{Q_{G}}{ND_{i}^{3}}\right),\;valid\;for\;\frac{Q_{G}}{ND_{i}^{3}}>0.037$$

P: Power consumption of ungassed stirring, kW; Q_G_: Total gas volumetric flow rate, m^3^/s; N: Stirring speed, s^−1^;

D_i_: Impeller diameter, 0.078 m; η_S_: Efficiency number of stirring, 0.65 [24].

(7) $$P=\;3f_{C}N_{P}\rho_{b}N^{3}\frac{D_{i}^{5}}{g}$$

P: Power consumption of ungassed stirring, kW; N_p_: Power number, 5.2 (Rushton impeller in turbulent regime, according to the Reynolds number);

ρ_b_: Broth density, kg/m^3^; g: Gravitational acceleration, m/s^2^.

(8) $$f_{C}=\;\sqrt{\frac{{\left(D_{R}/D_{i}\right)}^{ˆ}{\left(H_{L}/D_{i}\right)}^{ˆ}}{\left(D_{R}/D_{i}\right)\left(H_{L}/D_{i}\right)}}$$

D_R_: Bioreactor diameter, 0.160 m; H_L_: Height of the liquid, 0.267 m;

‘^’ indicates the geometric characteristics of the STR used in the experiments.

#### 2.2.4 Cost of cooling

In order to maintain a constant bioreactor temperature, it is necessary to remove the heat released by cell metabolism (Q_HEAT_) and the heat transferred from the impellers (E_S_) to the culture broth. A theoretical approach to estimate the cost of cooling was used, considering that all the heat generated by the cells and impellers was totally transferred to the cooling water, assuming a negligible lag phase duration. Hence, cooling of the broth by gas stripping and losses to the environment were neglected. The metabolic heat released during the bioreactor cultures was correlated to the oxygen uptake rate (OUR) using Eq. 9, proposed for aerobic microorganisms by Shuler and Kargi [61], as a generalization of the expressions reported by Cooney et al. [62] and Abbott and Clamen [63].

The OUR values were automatically calculated using Eq. 10, which was obtained from the nitrogen and oxygen molar balances in the gas phase, based on pressure (p), temperature (T), total volumetric gas flow (Q), and molar fractions of oxygen (O) and carbon dioxide (C) in the gas streams entering (1) and leaving (2) the bioreactor. The total metabolic energy generation (E_M_) was calculated by integration of Q_HEAT_ over time, up to the experimental point of maximum P_P_, and was added to the total E_S_ value obtained as described in the previous section. Next, the theoretical cost of cooling was estimated using Eq. 11, assuming an efficiency number (η_B_) of 0.7 [29].

(9) $${\;Q}_{HEAT}=\;K.OUR{.V}_{R}$$

K: Constant of proportionality, 0.50 kJ/mmol O_2_;

OUR: Oxygen uptake rate, mmol O_2_/(L.h);

Q_Heat_: Metabolic heat release rate, kJ/h.

(10) $$OUR=\;\frac{pQ_{1}}{RV_{R}}\left[y_{O1}-y_{O2}\frac{\left(1-y_{O1}\right)}{\left(1-y_{O2}-y_{C2}\right)}\right]$$

p = Pressure of entering gas, atm;

Q_1_ = Entering gas volumetric flow rate, L/h;

T_1_ = Temperature of entering gas, K;

R: Ideal gas constant, 8.2 × 10^−5^ atm.L/(mmol.K).

(11) $${\;\$}_{COOLING}=\;0.126\frac{\left(E_{M}+E_{S}\right)}{\eta_{B}}\frac{1}{V_{R}\left(C_{X\;}or\;P_{P}\right)}$$

$_COOLING_: Cost of cooling, US$/(g_DCW_ or g_PspA4Pro_); E_M_: Total metabolic energy generation, kWh;

η_B_: Efficiency number of thermostatic bath, 0.7 [29].

### 2.3 Statistical analysis

All the results presented are the average values for different experimental analyses carried out in triplicate and the propagation of their standard deviations. Genuine replicates were provided by Experiments #7.a and #7.b. The Tukey test was used to evaluate statistical differences [64], where p-values <0.05 were considered significant.

## 3 Results and discussion

### 3.1 PspA4Pro and biomass production costs: general aspects

**Fig. 1:**
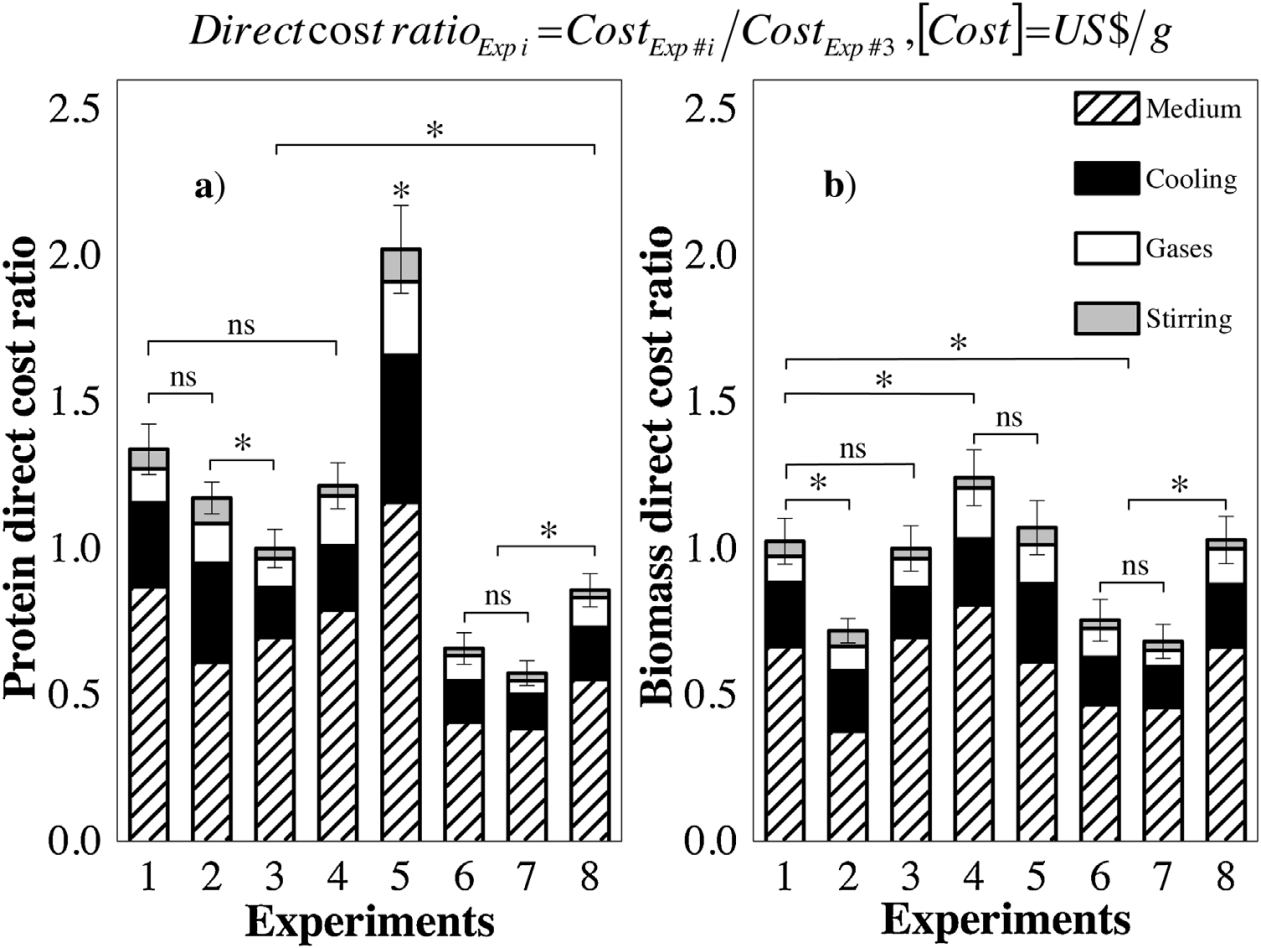
Direct cost ratios, using Experiment #3 as a reference (ref.), in terms of (a) PspA4Pro (ref. US$ 96.5/kg_PspA4Pro_) and (b) biomass (ref. US$ 11.9/kg_DCW_).*The Tukey test was performed to evaluate statistical differences, considering standard deviations and a p value <0.05 as significant. ns: not significant.

The medium and cooling, in that order, were the main components of the direct cost, regardless of the cultivation strategy used, representing more than 80 % of the direct cost for any experiment evaluated (Fig. 1). High contributions of the medium to the costs have been evidenced in other studies with recombinant proteins [25,31]. Initially, the complex media (Experiments #6, #7, and #8) provided the lowest direct PspA4Pro costs, in agreement with the findings of Zhang and Greasham [32] for low-cost products, but this would strongly depend on nitrogen source prices (as will be demonstrated in the last section). Experiments #6, #7, and #8, performed with complex media, presented approximately 70–80 % of the costs of Experiment #3, for which the PspA4Pro cost was the best value obtained using defined medium (Fig. 1.a). Higher soluble recombinant protein production and a shorter cultivation time using complex media (Table 1) seemed to be the main reasons for these results obtained during bioreactor batches, since prolonged protein expression could increase costs [28].

In the case of biomass production (Fig. 1.b), Experiments #2, #6, and #7 resulted in the lowest overall cost ratios, because the higher biomass concentrations contributed to mitigating the costs (Table 1). For the other cultivation strategies investigated, the differences in the overall cost ratio values were not statistically significant.

### 3.2 PspA4Pro and biomass production costs: specific aspects

#### 3.2.1 Effect of temperature on direct cost

Operation at moderate temperature appeared to reduce the cost of PspA4Pro production. Comparison of the results for Experiments #1 (27 °C), #3 (32 °C), and #4 (37 °C) (Fig. 1), which were all performed with defined medium and induction by IPTG (Table 1), showed that the lowest cost in terms of protein was obtained for Experiment #3. For this condition, a better balance between metabolic burden and biomass production was likely to reduce the cost [38,65]. This possibility was reinforced by the results of Experiments #2 and #5 (Table 1), since less protein was produced at 37 °C than at 27 °C, possibly as a consequence of a higher metabolic burden on the host cells in the former experiment [66,67], which could be partially evidenced by high plasmid losses during Experiment #5 at 37 °C [47].

#### 3.2.2 Effect of inducers on energy costs

**Fig. 2:**
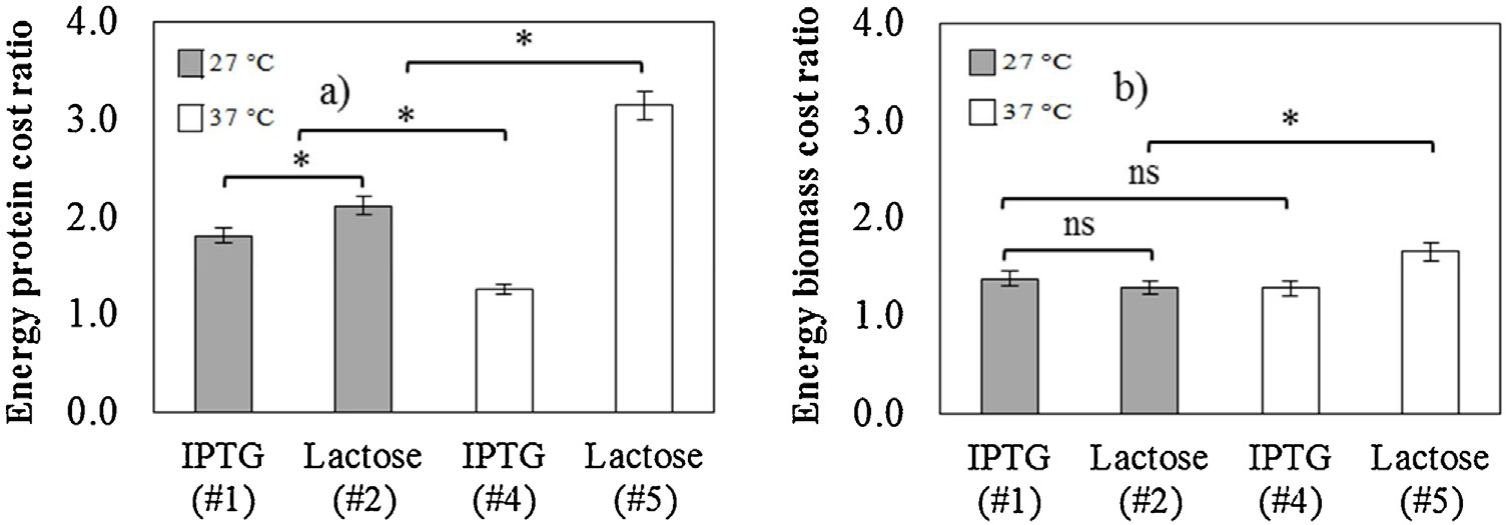
Effect of inducers on energy costs (cooling, stirring, and compressor operations). Cost ratios* in terms of (a) PspA4Pro (ref. US$ 21.9/kg PspA4Pro) and (b) biomass (ref. US$ 2.7/kg DCW). All experiments using defined medium. *Reference: Experiment #3 (Table 1).*The Tukey test was performed to evaluate statistical differences, considering standard deviations and a p value <0.05 as significant. ns: not significant.

The type of inducer can influence the PspA4Pro cost in terms of the energy required during bioreactor batches. The protein costs related to energy consumption (for cooling, stirring, and compressor operations) were lower when IPTG was used as the inducer, rather than lactose, at the same temperatures (Fig. 2). Compared to lactose, the use of IPTG as inducer leads to faster protein synthesis [28,67], with the higher PspA4Pro production resulting in shorter induction phases, hence reducing energy costs. Generally, faster recombinant protein synthesis has been associated with an increase in inclusion bodies or low quality recombinant protein (incorrectly folded, or with impaired function and properties) [68,69]. In fact, due to their coiled-coil α-helix structure [46], N-terminal fragments of PspA are very soluble and thermostable. When PspA4Pro was heated from 15 to 95 °C, followed by cooling to 15 °C, it recovered the original structure, as measured by CD, and a relatively thermostable region was identified between 40 and 50 °C [37]. In addition, the presence of PspA4Pro in inclusion bodies was never found in the culture samples analyzed (Figure S2). For these reasons, fast induction strategies can be applied during *E. coli* cultures to produce PspA, without affecting its secondary structure (Figure S3). Thus, the most suitable induction strategy depends on the protein characteristics and should be investigated on a case-by-case basis.

#### 3.2.3 Influence of component price on medium cost

##### 3.2.3.1 Complex medium (auto-induction)

**Fig. 3:**
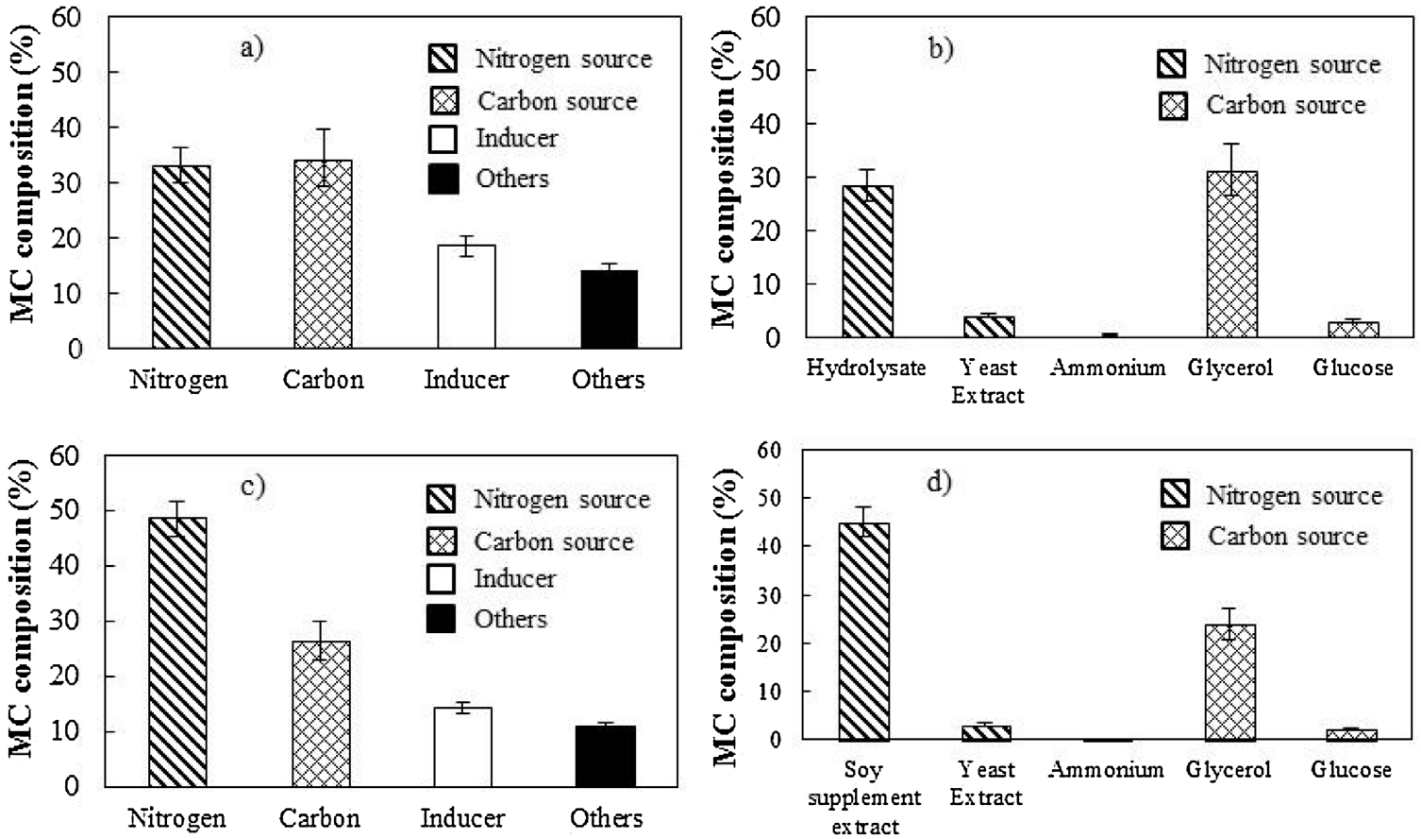
Cost of complex medium with peptones: (a) core costs; (b) nitrogen and carbon sources in detail. Cost of complex medium with soy supplement: (c) core costs; (d) nitrogen and carbon sources in detail. MC: medium cost; Suppl. Ext: supplement extract.

For the cost estimation based on the information obtained from business intelligence and e-commerce platforms [56,57], the nitrogen and carbon sources were major contributors to the costs in Experiments #6, #7, and #8, performed according to the auto-induction strategy, with lactose as inducer. These components represented around 70 % of the medium cost (Fig. 3.a and 3.c), while the inducer (lactose) and other medium components played less important roles. Considering all the nitrogen sources, the protein hydrolysates (ANS or VNS) had the greatest impact on the cost (Fig. 3.b), because their prices were approximately 3 times higher than the market value of yeast extract, which was also present in the medium with half the hydrolysate concentration (Table 2).

**Fig. 4:**
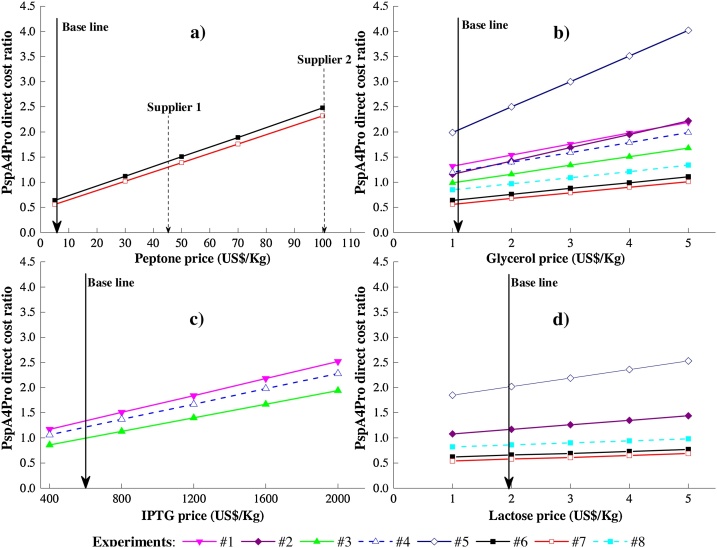
Sensitivity analyses of peptone, glycerol, IPTG, and lactose prices on PspA4Pro direct cost. Survey involving certified peptones from three large market suppliers. Reference: Experiment #3 (US$ 96.5/kg PspA).

The impact of the nitrogen source prices on costs could be even greater, given that ANS and VNS prices can vary widely according to the supplier. For example, when peptone was replaced by soybean protein supplement extract in Experiment #8, adopting its current sale price, there was an increase of 15 % in the contribution of the nitrogen source to the cost of the medium (Fig. 3.d). According to sensitivity analysis of peptone prices (Fig. 4.a), if the commercial certified peptone with the best price (US$ 45/kg) found in our survey was considered in the calculations (Supplier 1), it would lead to an increase of 125 % in the PspA4Pro cost, relative to the value estimated using the commercial platform prices. Similarly, if the second best supplier was considered, it would lead to an increase of around 400 %. As shown in Fig. 4.a, the certified peptone prices of different market suppliers strongly impacted the direct cost. In the same way, the glycerol price had some impact on the PspA4Pro direct cost (Fig. 4.b). However, this raw material may not offer a great opportunity to decrease the production cost, since as a commodity [70,71], it is commonly offered by several suppliers at lower prices than those of nitrogen sources. Hence, these findings indicate that nitrogen sources are more relevant than carbon sources, in terms of the costs of cultivation using complex media, and therefore represent a potential target for lowering the costs of recombinant protein and biomass production.

Comparison of Experiments #6, #7, and #8 (Fig. 1) showed that the use of nitrogen from animal or vegetal sources led to low costs for producing PspA4Pro. However, for biopharmaceutical production, peptones from vegetal sources seem to be more suitable, since they contain prion-free protein and are in compliance with good manufacturing practices (GMP). Tryptones with prion-free certification are also available and can be used for biopharmaceuticals production, but they are more expensive than ordinary enzymatic casein hydrolysates. As discussed in the next section, the prices of peptones may be crucial criteria in terms of medium selection.

##### 3.2.3.2 Defined medium

**Fig. 5:**
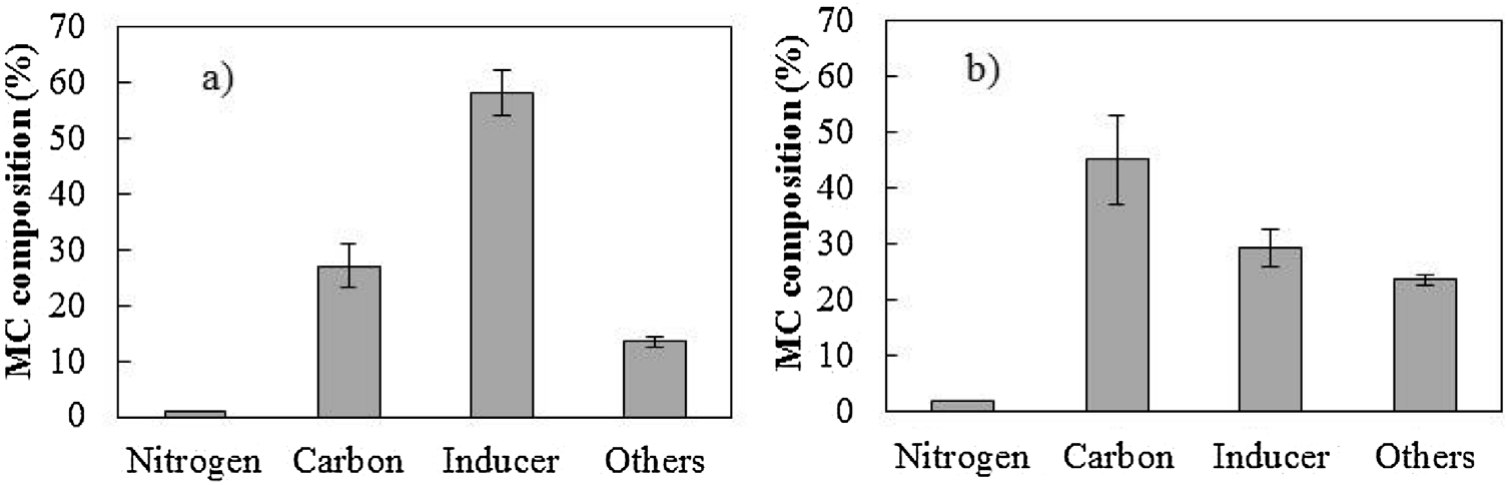
Defined medium costs. Batch cultivations using (a) IPTG and (b) lactose. MC: medium cost.

Concerning the cultivations carried out with defined medium (Experiments #1-#5, Table 1), the choice of inducer plays an important role in determining costs [32]. When IPTG was used as the inducer in the cultivations, this compound presented the highest percentage of the medium cost, with a contribution 2-fold higher than that of glycerol, the sole carbon source used (Fig. 5.a). Although the amount of IPTG added to induce PspA4Pro expression was very small (1 mM), it was by far the most expensive component (Table 2). Since lactose is cheaper than IPTG, when it replaced IPTG as the inducer in the cultivations, glycerol became the most relevant medium component in terms of cost (46 %), followed by lactose (30 %) (Fig. 5.b). Thus, the prices of IPTG, glycerol, and lactose may have substantial effects on the direct costs of PspA4Pro production using defined medium (Figs. 4.b, 4c, and 4d). It is important to emphasize that defined medium formulations also comply with GMP guidelines [32], even using IPTG as inducer, since the presence of this substance as a contaminant in the final product is very unlikely, due to the extensive purification steps used to obtain biopharmaceuticals [37].

### 3.3 Influence of cultivation strategy on PspA4Pro quality

The quality of the protein produced is a crucial issue to be considered when choosing the cultivation strategy, since the biological function of the recombinant protein could be affected by cultivation conditions [68]. The analysis of inclusion bodies formation showed that, irrespective of the cultivation strategy adopted, no PspA4Pro was aggregated and accumulated as insoluble inclusion bodies (Figure S2), suggesting that PspA4Pro was correctly folded in all cases. However, PspA has no enzymatic activity, so it is not possible to evaluate its biological function (lactoferrin binding capability) without previous protein purification. Similarly, the analysis of secondary structure by CD can only be performed with pure protein samples. Therefore, four different cultivation conditions were selected for protein purification, in order to evaluate the influence of the protein production strategy on PspA4Pro quality: Experiment #2, using defined medium and mild induction conditions; Experiment #3, which was the most cost-effective cultivation strategy; Experiment #4, using defined medium and with fast biomass growth; and Experiment #8, which was carried out with the alternative nitrogen source (Table 1).

The purification of PspA4Pro from the biomass harvested in the selected experiments was performed following the procedures described previously [37], with 96–98 % final purities obtained for all the purification processes performed. The results of CD analyses and lactoferrin binding assays for Experiments #2, #3, #4, and #8 are provided in the Supplementary Material. The results for Experiment #7 were reported by Figueiredo et al. [37]. The CD spectra demonstrated that PspA4Pro had the expected secondary structure in all cases (using different media, temperatures, and protein syntheses), being comparable to the PspA4Pro standard as well as to literature data [45,46,72,73], presenting two typical valleys with minima at 208 nm and 222 nm.

The PspA4Pro purified from all four experiments showed equal ability to bind lactoferrin, with the same slope for binding according to PspA4Pro concentration, indicating that the product of each experiment had the same quality (Figure S3). In addition, the lactoferrin binding assay also indicated that rabbit anti-PspA4Pro antibodies, which bind to the PspA4Pro attached to lactoferrin bound to the plate, recognized PspA4Pro from all the experiments.

### 3.4 PspA4Pro protein and biomass production costs: points to highlight

After performing the economic analysis of the production of PspA4Pro using *E. coli* BL21(DE3) in the bioreactor, according to the process factors assessed in this work, the most important points to consider in the selection of a suitable cultivation strategy were identified, as discussed below.

There is a limit value for the price of nitrogen sources that determines whether defined or auto-induction complex media should be selected for the production of the protein at low cost. In order to find this threshold value, the peptone price in Experiment #7 (Table 1) was varied so that the PspA4Pro direct cost ratio was equivalent to the best performance achieved with a defined medium (Experiment #3), considering all other raw material prices and process parameters to remain constant. Following the criteria adopted during the present cost analysis, the threshold price obtained was about US$ 30/kg for ANS/VNS, which could be used to decide when to use complex or defined medium during a bioreactor batch. However, commercial peptone products with prion-free certifications are commonly sold for prices above this threshold value (Fig. 4), different from the e-commerce platform peptone prices used in the present cost estimates. Furthermore, the survey of different ANS and VNS suppliers showed that the prices can vary significantly according to the manufacturer, quality, and quantity purchased, with the minimum price of soy peptone being US$ 45/kg (1.5-fold higher than the threshold price). Therefore, the results demonstrated that in this case, use of a defined medium was the most cost-effective option.

Efforts to provide low-cost raw materials with acceptable quality are required in order to broaden the usage of complex media in r*E. coli* cultures. The “homemade” extract of soy protein supplement for human consumption used in Experiment #8 is an example of a cheap nutrient that can lead to a competitive direct cost. It is also important to remember that for bio-based products such as industrial enzymes and chemicals, ordinary ANS/VNS can be employed in r*E. coli* cultures [28], whereas certified nitrogen sources are a specific requirement for biopharmaceutical production, which can make protein production more expensive. Moreover, the quality of the nutrients used for medium formulation may have different effects on synthesis of a specific heterologous protein, affecting its molecular integrity and leading to inclusion body formation [74]. Therefore, quality assessment of the final product should be provided when non-conventional raw material sources or undefined materials (such as peptones or yeast extract, among others) from different suppliers are used, in order to guarantee protein function and properties. For this reason, it is always important to perform specific studies to evaluate commercial peptones and yeast extract from different suppliers, in order to assess the impacts of these nutrients on process economics and product quality.

Care is needed in selection of the temperature used to cultivate *E. coli* cells and produce soluble recombinant proteins. Although PspA4Pro presented the best production results at 32 °C, other heterologous proteins would be unlikely to show the same behavior [35]. Metabolic burden, formation of inclusion bodies, and protein quality impairment are some important factors to evaluate for each different recombinant product [68]. Studies have indicated that low temperature seems to help in avoiding these issues, or at least in mitigating them during *E. coli* cultivations [75,76]. However, these results did not consider cost impacts on processes. Studies with bioreactor cultivations may be time consuming and expensive in terms of the production, purification, and analysis of recombinant products. They may also lead to a trial-and-error approach, because several factors may influence this type of production [47]. Nonetheless, efforts aiming at cost-effective recombinant protein production should be encouraged, in order to improve decision-making before implementing process strategies.

According to the findings of the present economic analysis, induction with IPTG seemed to be the best strategy for production of PspA4Pro. As discussed previously, induction with IPTG appeared to intensify and accelerate recombinant protein production, resulting in lower process costs. It is clear that the choice of inducer (as well as the induction strategy) must be evaluated on a case-by-case basis, according to the characteristics of the target recombinant protein. Nonetheless, this contradicts the general opinion that induction with IPTG is not industrially viable, due to its high price [27,65,77,78]. In fact, considering only the impact of the medium on the cost of recombinant protein or biomass production, IPTG would be the most expensive raw material. However, taking the comprehensive approach of the present cost analysis for PspA4Pro production, IPTG would be the best choice. In addition, the IPTG concentration could be reduced [27,79,80], further decreasing the production cost of this recombinant protein.

Besides the application of the proposed methodology to identify cultivation conditions for PspA4Pro production, as discussed above, there are some considerations concerning extending the economic analysis approach developed here to other situations. Although there are several different culture media and bioreactor operational modes that can be employed to produce recombinant proteins in *E. coli*, the results observed for PspA4Pro may be valid when the recombinant protein solubility is not (or minimally) affected by the culture conditions. In fact, there are many recombinant antigens reported as fully soluble when produced in recombinant *E. coli* using Lac operon expression systems: pneumococcal proteins such as ZmpB [81], neuraminidase A [82], and PotD [83]; autolysin from *Listeria monocytogenes* and *Pseudomonas aeruginosa* [84,85]; transferrin binding proteins A from *N. meningitidis* [86]; hyaluronate lyase and PspC from *Streptococcus suis* [87,88]; and the cholesterol-dependent cytolysin family, including streptolysin O, pneumolysin, suilysin [89], and arcanolysin [90]. In common with PspA, all these molecules are single-chain polypeptides without disulfide bonds.

Even though the influence of the operational conditions on the process economics was based on bench-scale STR bioreactor data, the proposed approach may be applied for evaluating different STR bioreactor scales, including the larger ones present in the industry, since the theoretical equations used are general. Besides, the methodology may be easily extended to air-lift and similar bioreactors simply by setting the stirring speed to zero in such cases. Furthermore, the concept of “direct cost ratio” should be mentioned. It was introduced to make the economic analysis general and less dependent of the bioreactor scale as well as of unavoidable fluctuations in the prices of medium components and energy. On the other hand, if desired, the cost analysis can be also based on the actual prices of raw-materials and utilities known for a specific cultivation case and lead to the identification of the main targets for a lower cost operation.

In addition to recombinant proteins, *E. coli* can be used to synthesize value-added biomolecules such as amino acids, organic acids, and advanced biofuels, corresponding to a global trade of US$ 22 billion [[91], [92], [93]]. Using systems biology tools, this bacterium has strong potential to become the future microbial factory for sustainable production of bio-based chemicals, especially considering its ability to grow in inexpensive, abundant, and renewable feedstocks, including lignocellulosic biomass hydrolysates [94] and crude glycerol generated from biodiesel production [95]. The calculation procedure developed here could also be used to assess the economics of the production of these bio-based chemicals, as well as to identify the most favorable bioreactor cultivation conditions.

It is also important to point out that the outlined methodology is suitable for estimation of the direct costs of the cultivation process. The real cost of a recombinant protein (or any bioproduct) depends on the overall production cost, which encompasses all steps in upstream and downstream processing and accounts for equipment depreciation, capital cost, and labor cost, in addition to the direct production costs.

## 4 Conclusions

Based on usual cost estimation equations for cultivation supplies and energy consumption, combined with design correlations, the approach developed here was applied in the assessment of process economics using data from 8 different bioreactor process strategies. In the case study discussed here, the best strategy was characterized by a short induction phase, carried out at moderate temperature (32 °C) and with IPTG as inducer, in order to maintain high PspA4Pro production rates, together with lower cell metabolic stress and reduced energy consumption.

The prices of nitrogen sources used in complex media seem to be determinant for selection of the type of media, especially when it is necessary to ensure adherence to GMP standards for biopharmaceutical production, as in the case of PspA4Pro. On the other hand, ordinary ANS/VNS, such as soy supplement, may face some constraints due to this regulatory issue, although such materials may be suitable for the production of bio-based enzymes and chemicals. In general, for certified commercial peptones with prices above US$ 30/kg, defined medium is the most cost-effective choice. However, the use of complex media is a promising approach for reducing the production cost of the target protein, given that research efforts are made to address critical issues, such as the characterization of peptones from different sources and with different qualities and prices, in terms of nutrient content, and their impacts on product and biomass yields. Furthermore, it is also necessary to ensure the commercial availability of certified peptones with more accessible prices.

Selection of the best cultivation strategy clearly depends on the target product, host cell, and production scale, requiring analyses on a case-by-case basis. However, provided that basic information about process conditions, biomass concentration, and product formation is available, the theoretical approach presented here to analyze the effects of different media, inducers, and complex nitrogen sources on PspA4Pro production can be easily extended to evaluation of the economics of any bioreactor cultivation, contributing to identification of the most cost-effective strategy.

## Author contributions

(1) the conception and design of the study, or acquisition of data, or analysis and interpretation of data, (2) drafting the article or revising it critically for important intellectual content, (3) final approval of the version to be submitted.

## CRediT authorship contribution statement

**Valdemir M. Cardoso:** Conceptualization, Methodology, Investigation, Validation, Data curation, Visualization, Writing - original draft, Formal analysis. **Gilson Campani:** Investigation. **Maurício P. Santos:** Investigation. **Gabriel G. Silva:** Investigation. **Manuella C. Pires:** Investigation. **Viviane M. Gonçalves:** Supervision, Writing - review & editing, Funding acquisition. **Roberto de C. Giordano:** Supervision, Funding acquisition. **Cíntia R. Sargo:** Investigation. **AntônioC.L. Horta:** Investigation, Software. **Teresa C. Zangirolami:** Conceptualization, Methodology, Validation, Writing - review & editing, Supervision, Funding acquisition.

## Declaration of Competing Interest

The authors declare that they have no known competing financial interests or personal relationships that could have appeared to influence the work reported in this paper.
