# Supplementary material for: Cost analysis based on bioreactor cultivation conditions: Production of a soluble recombinant protein using Escherichia coli BL21(DE3)
Source: Biotechnol Rep (Amst). 2020 Feb 22;26:e00441. doi: 10.1016/j.btre.2020.e00441 (PMC7049567; doi:10.1016/j.btre.2020.e00441)
Supplement: Supplementary file 2 [file mmc2.pptx]

## Slide 1
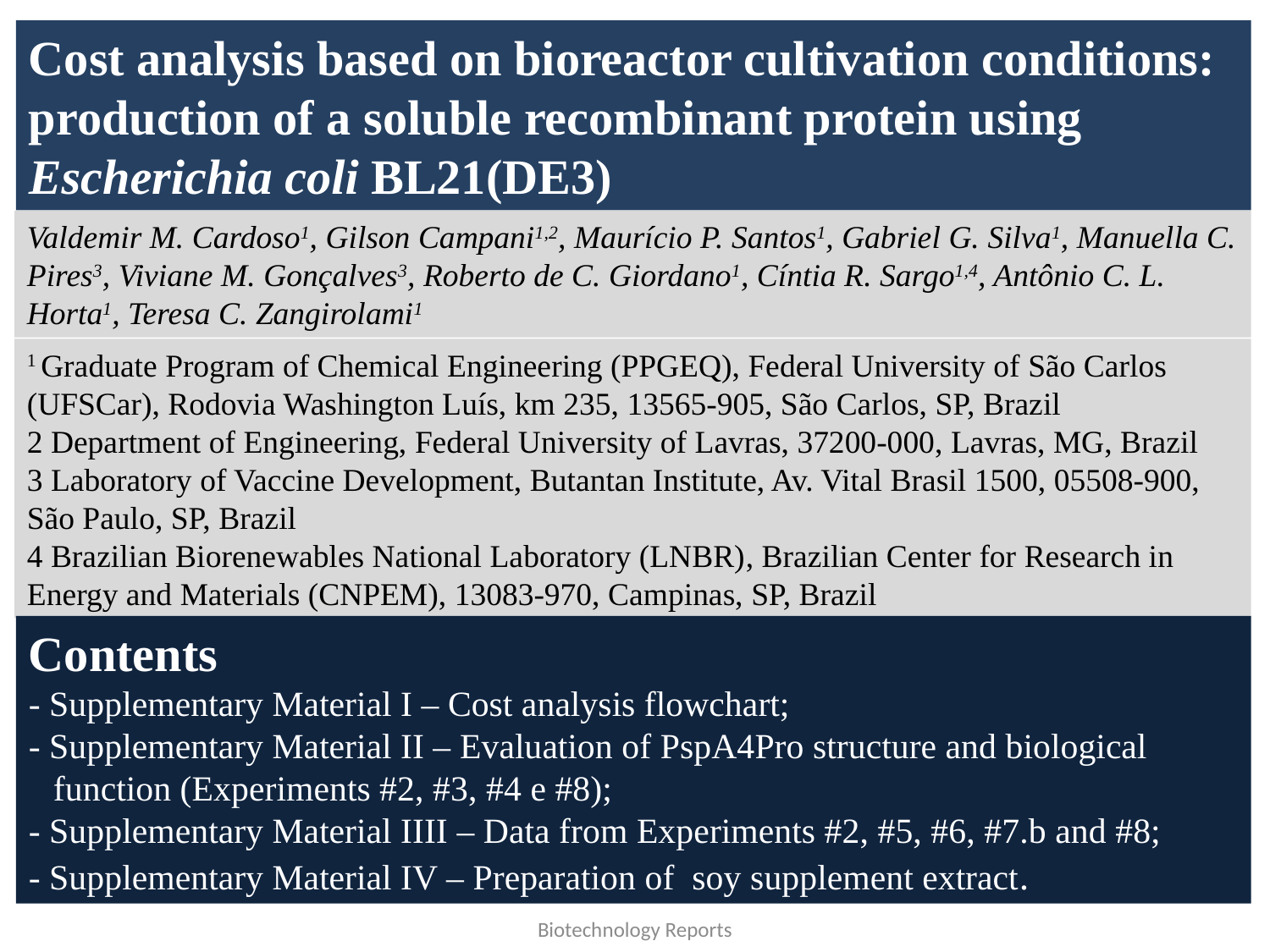

Cost analysis based on bioreactor cultivation conditions: production of a soluble recombinant protein using Escherichia coli BL21(DE3)
Valdemir M. Cardoso1, Gilson Campani1,2, Maurício P. Santos1, Gabriel G. Silva1, Manuella C. Pires3, Viviane M. Gonçalves3, Roberto de C. Giordano1, Cíntia R. Sargo1,4, Antônio C. L. Horta1, Teresa C. Zangirolami1
1 Graduate Program of Chemical Engineering (PPGEQ), Federal University of São Carlos (UFSCar), Rodovia Washington Luís, km 235, 13565-905, São Carlos, SP, Brazil
2 Department of Engineering, Federal University of Lavras, 37200-000, Lavras, MG, Brazil
3 Laboratory of Vaccine Development, Butantan Institute, Av. Vital Brasil 1500, 05508-900, São Paulo, SP, Brazil
4 Brazilian Biorenewables National Laboratory (LNBR), Brazilian Center for Research in Energy and Materials (CNPEM), 13083-970, Campinas, SP, Brazil
Contents
- Supplementary Material I – Cost analysis flowchart;
- Supplementary Material II – Evaluation of PspA4Pro structure and biological function (Experiments #2, #3, #4 e #8);
- Supplementary Material IIII – Data from Experiments #2, #5, #6, #7.b and #8;
- Supplementary Material IV – Preparation of soy supplement extract.
Biotechnology Reports

## Slide 2
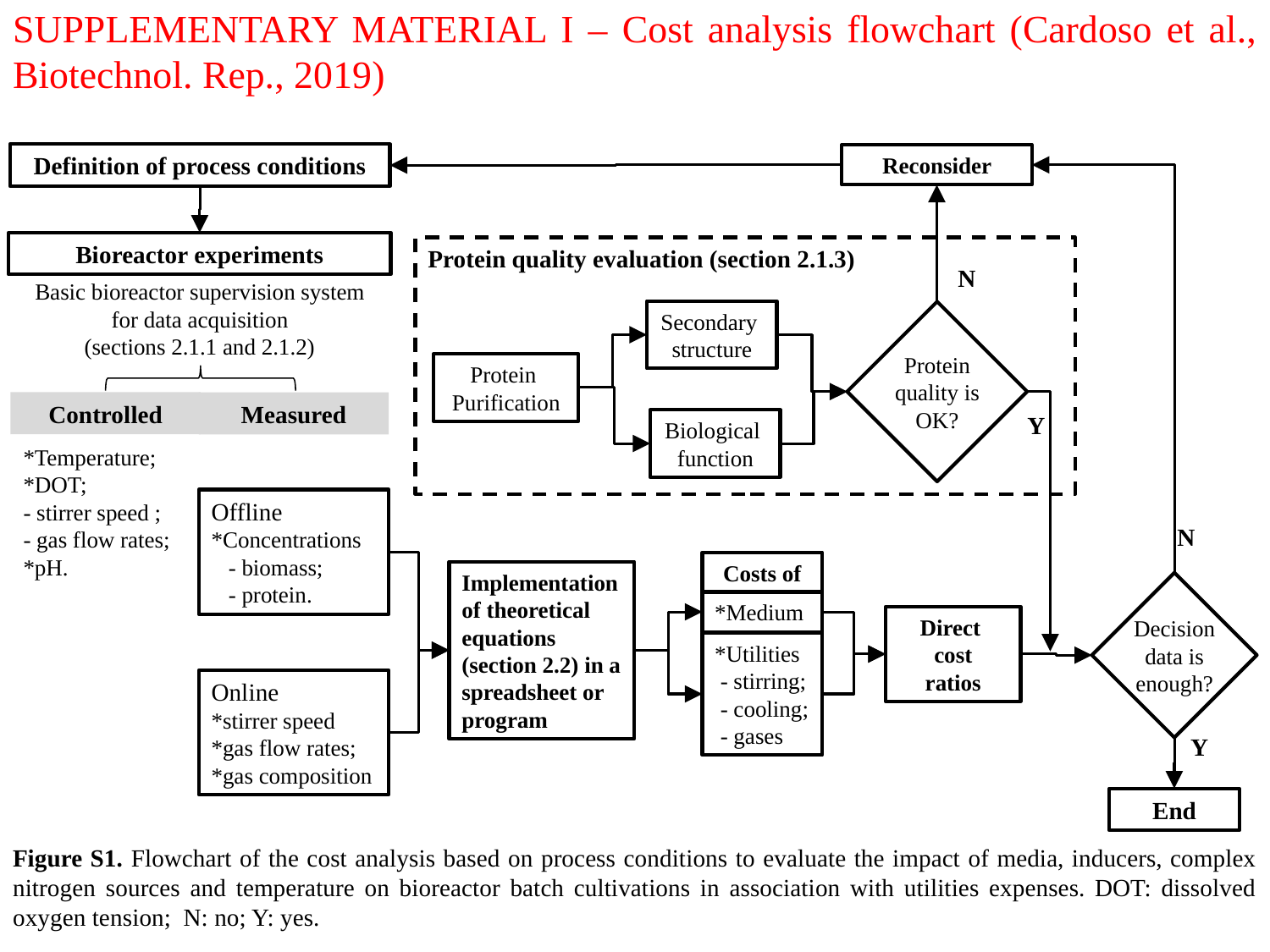

SUPPLEMENTARY MATERIAL I – Cost analysis flowchart (Cardoso et al., Biotechnol. Rep., 2019)
Definition of process conditions
Reconsider
Bioreactor experiments
Protein quality evaluation (section 2.1.3)
N
Basic bioreactor supervision system for data acquisition
(sections 2.1.1 and 2.1.2)
Secondary
structure
Protein quality is OK?
Protein
Purification
Controlled
Measured
Y
Biological
function
*Temperature;
*DOT;
- stirrer speed ;
- gas flow rates;
*pH.
Offline
*Concentrations
 - biomass;
 - protein.
N
Costs of
Implementation of theoretical equations (section 2.2) in a spreadsheet or program
Decision data is enough?
*Medium
Direct
cost
ratios
*Utilities
 - stirring;
 - cooling;
 - gases
Online
*stirrer speed
*gas flow rates;
*gas composition
Y
End
Figure S1. Flowchart of the cost analysis based on process conditions to evaluate the impact of media, inducers, complex nitrogen sources and temperature on bioreactor batch cultivations in association with utilities expenses. DOT: dissolved oxygen tension; N: no; Y: yes.

## Slide 3
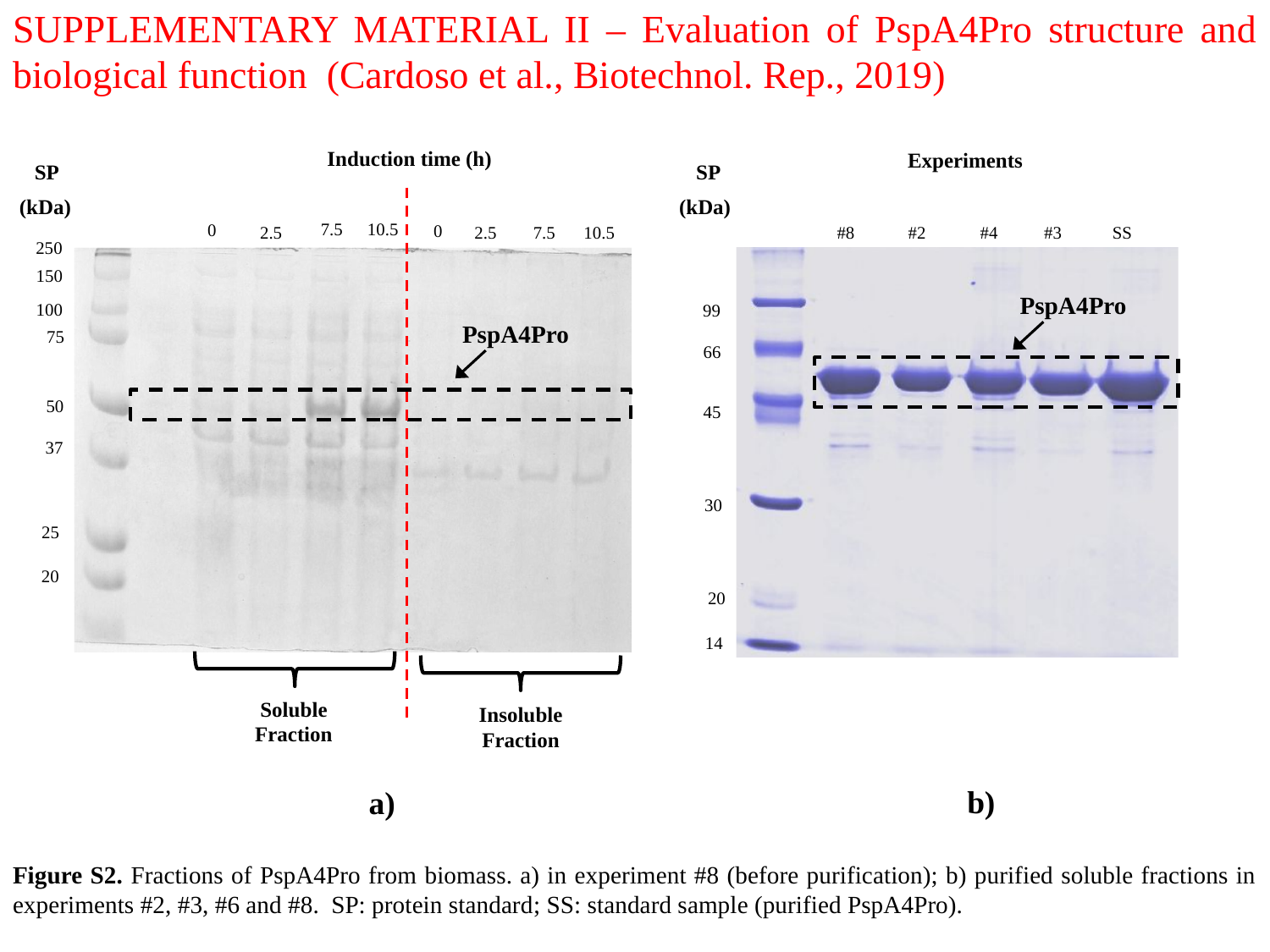

SUPPLEMENTARY MATERIAL II – Evaluation of PspA4Pro structure and biological function (Cardoso et al., Biotechnol. Rep., 2019)
Induction time (h)
Experiments
SP
SP
(kDa)
(kDa)
7.5
10.5
0
0
2.5
#2
SS
#8
2.5
#4
#3
7.5
10.5
250
150
 PspA4Pro
100
99
 PspA4Pro
75
66
50
45
37
30
25
20
20
14
Soluble
Fraction
Insoluble
Fraction
b)
a)
Figure S2. Fractions of PspA4Pro from biomass. a) in experiment #8 (before purification); b) purified soluble fractions in experiments #2, #3, #6 and #8. SP: protein standard; SS: standard sample (purified PspA4Pro).

## Slide 4
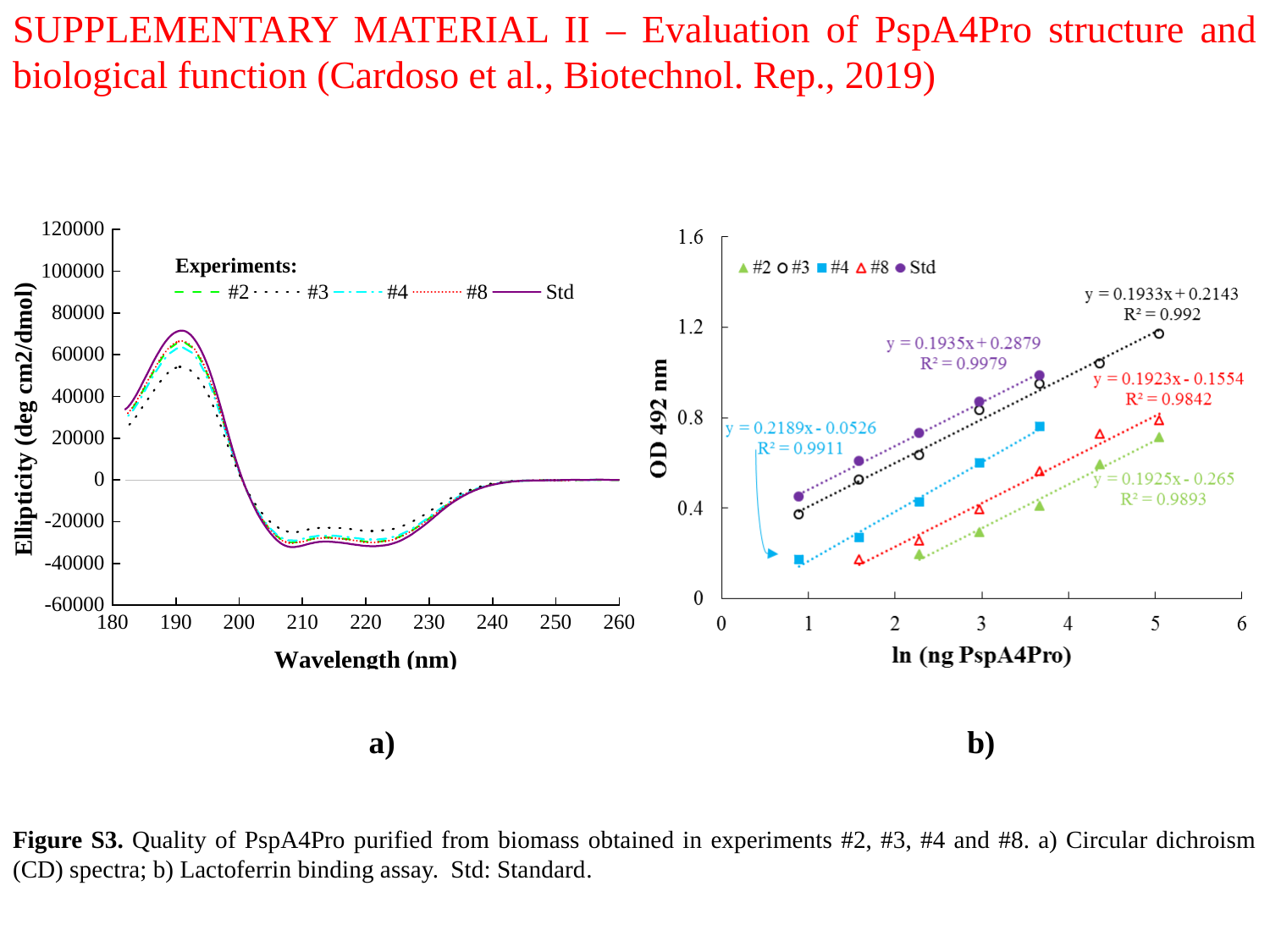

SUPPLEMENTARY MATERIAL II – Evaluation of PspA4Pro structure and biological function (Cardoso et al., Biotechnol. Rep., 2019)
b)
a)
Figure S3. Quality of PspA4Pro purified from biomass obtained in experiments #2, #3, #4 and #8. a) Circular dichroism (CD) spectra; b) Lactoferrin binding assay. Std: Standard.

## Slide 5
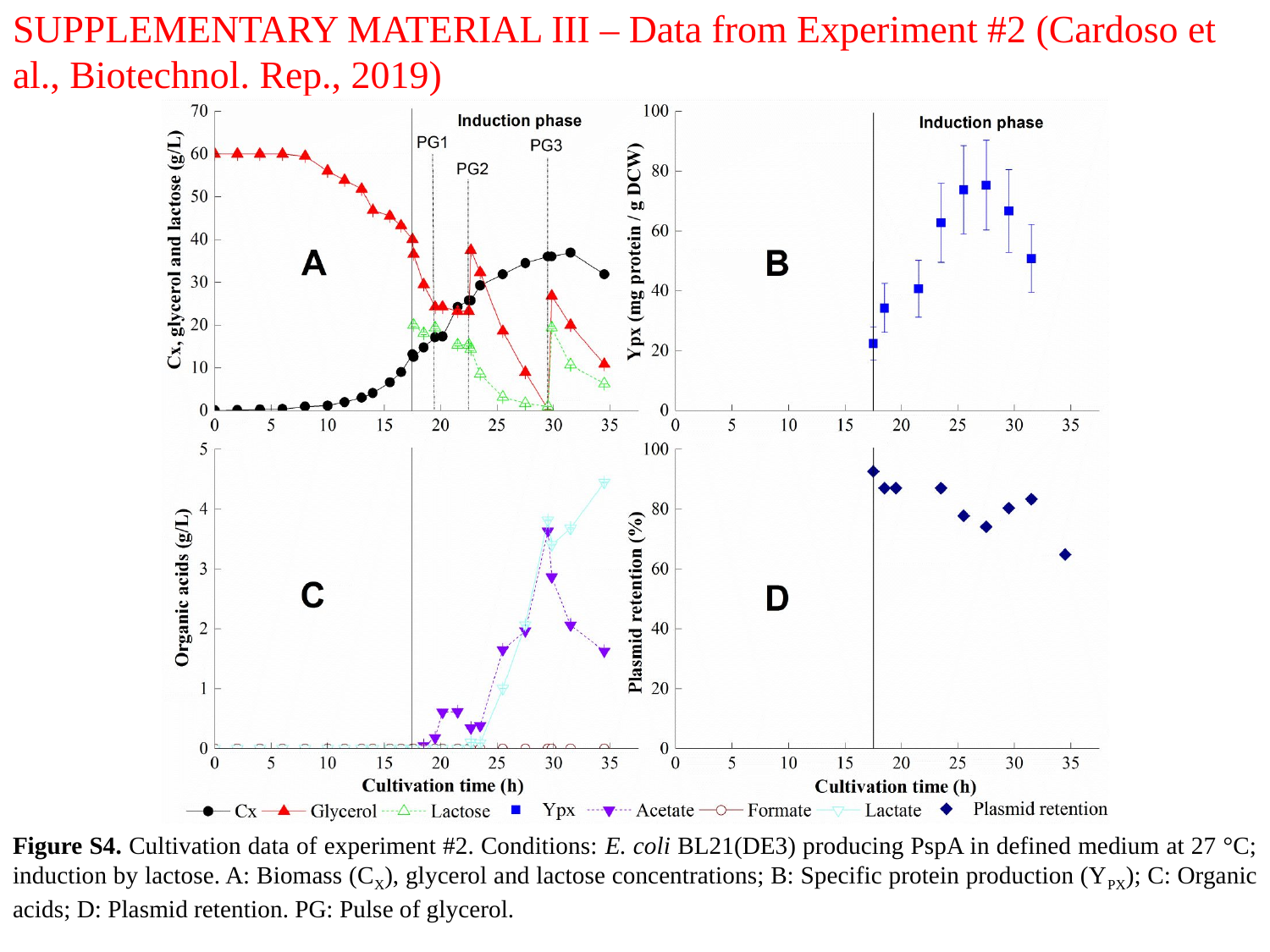

# SUPPLEMENTARY MATERIAL III – Data from Experiment #2 (Cardoso et al., Biotechnol. Rep., 2019)
Figure S4. Cultivation data of experiment #2. Conditions: E. coli BL21(DE3) producing PspA in defined medium at 27 °C; induction by lactose. A: Biomass (CX), glycerol and lactose concentrations; B: Specific protein production (YPX); C: Organic acids; D: Plasmid retention. PG: Pulse of glycerol.

## Slide 6
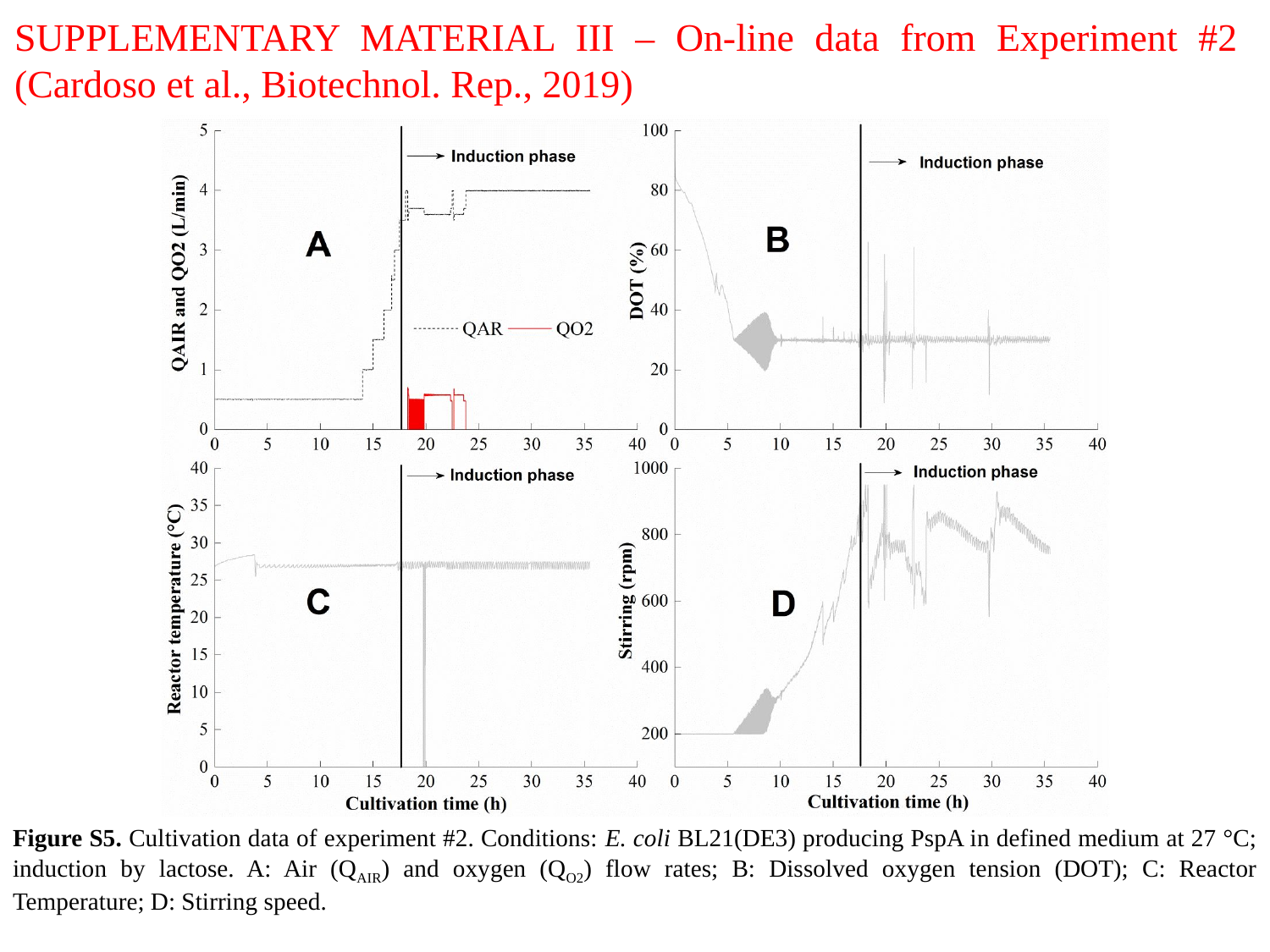

SUPPLEMENTARY MATERIAL III – On-line data from Experiment #2 (Cardoso et al., Biotechnol. Rep., 2019)
Figure S5. Cultivation data of experiment #2. Conditions: E. coli BL21(DE3) producing PspA in defined medium at 27 °C; induction by lactose. A: Air (QAIR) and oxygen (QO2) flow rates; B: Dissolved oxygen tension (DOT); C: Reactor Temperature; D: Stirring speed.

## Slide 7
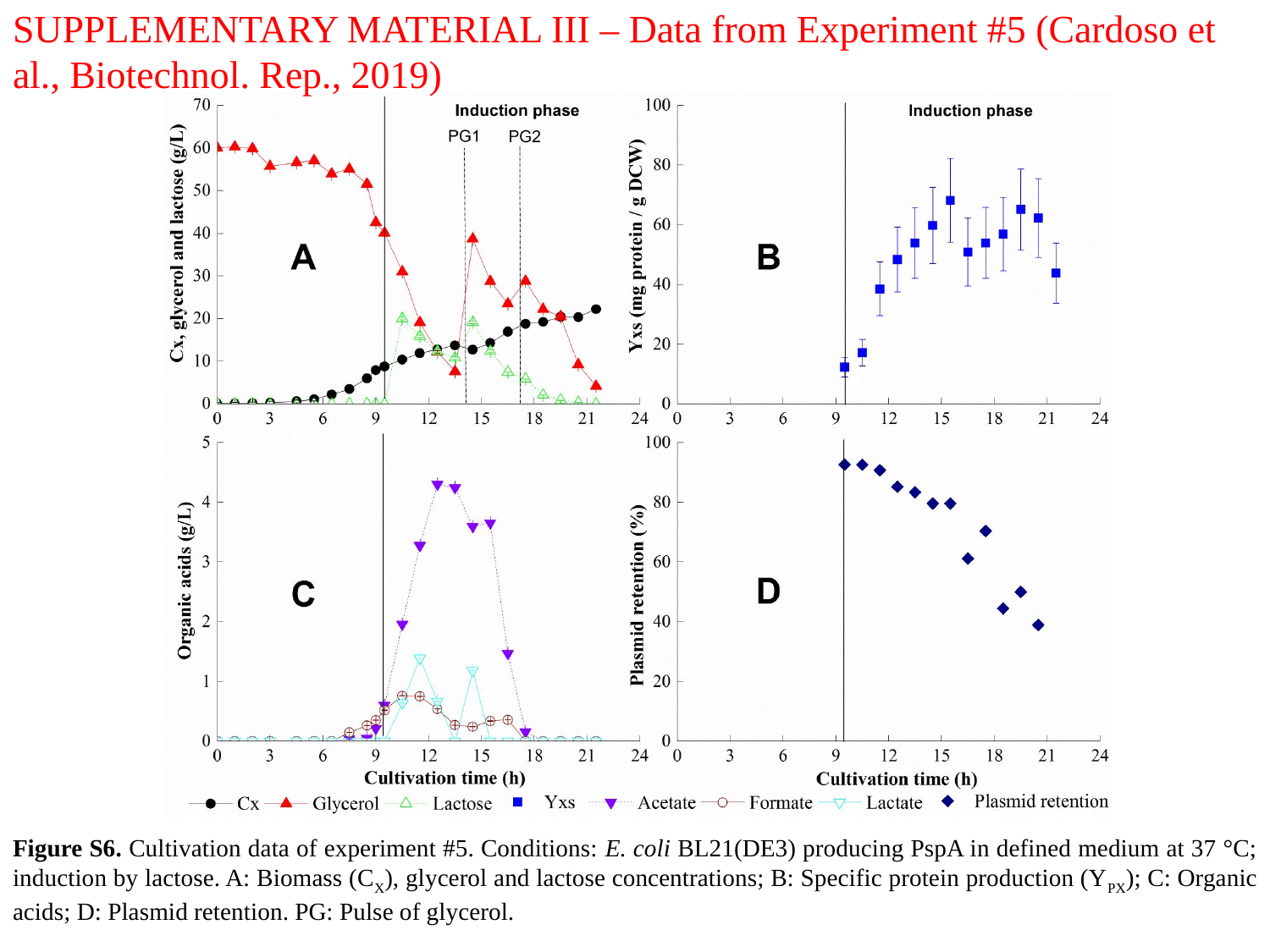

SUPPLEMENTARY MATERIAL III – Data from Experiment #5 (Cardoso et al., Biotechnol. Rep., 2019)
Figure S6. Cultivation data of experiment #5. Conditions: E. coli BL21(DE3) producing PspA in defined medium at 37 °C; induction by lactose. A: Biomass (CX), glycerol and lactose concentrations; B: Specific protein production (YPX); C: Organic acids; D: Plasmid retention. PG: Pulse of glycerol.

## Slide 8
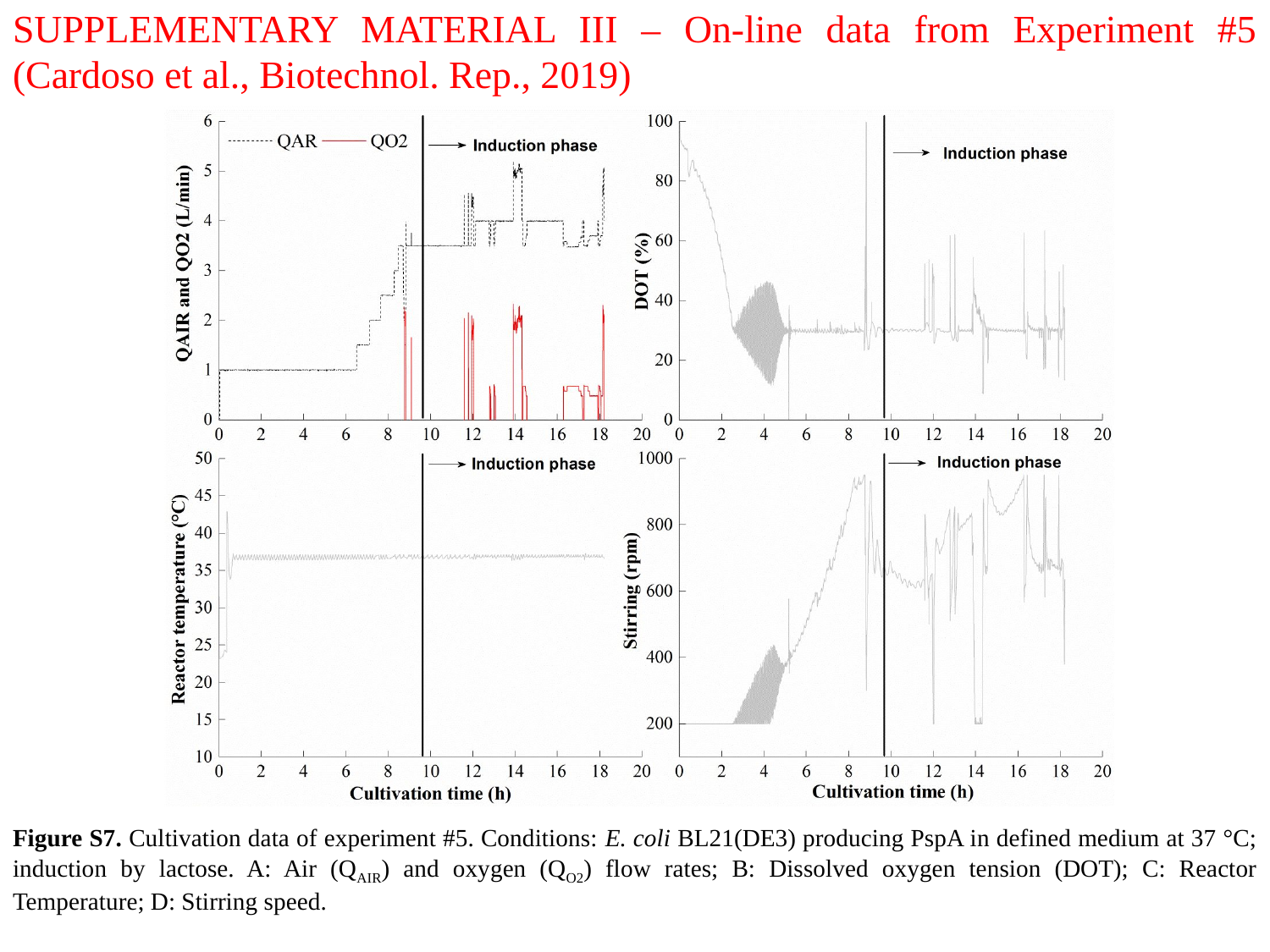

SUPPLEMENTARY MATERIAL III – On-line data from Experiment #5 (Cardoso et al., Biotechnol. Rep., 2019)
Figure S7. Cultivation data of experiment #5. Conditions: E. coli BL21(DE3) producing PspA in defined medium at 37 °C; induction by lactose. A: Air (QAIR) and oxygen (QO2) flow rates; B: Dissolved oxygen tension (DOT); C: Reactor Temperature; D: Stirring speed.

## Slide 9
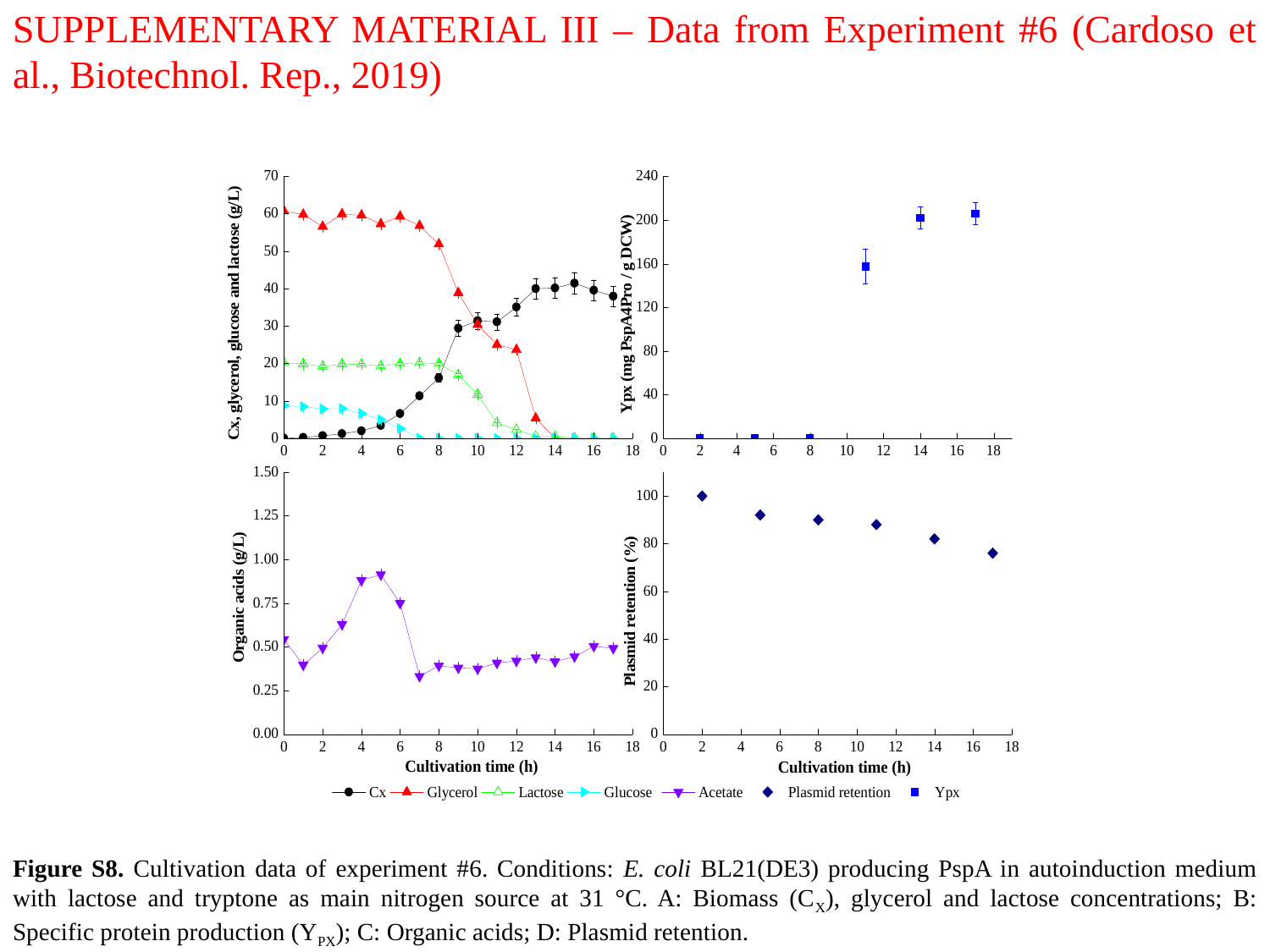

SUPPLEMENTARY MATERIAL III – Data from Experiment #6 (Cardoso et al., Biotechnol. Rep., 2019)
Figure S8. Cultivation data of experiment #6. Conditions: E. coli BL21(DE3) producing PspA in autoinduction medium with lactose and tryptone as main nitrogen source at 31 °C. A: Biomass (CX), glycerol and lactose concentrations; B: Specific protein production (YPX); C: Organic acids; D: Plasmid retention.

## Slide 10
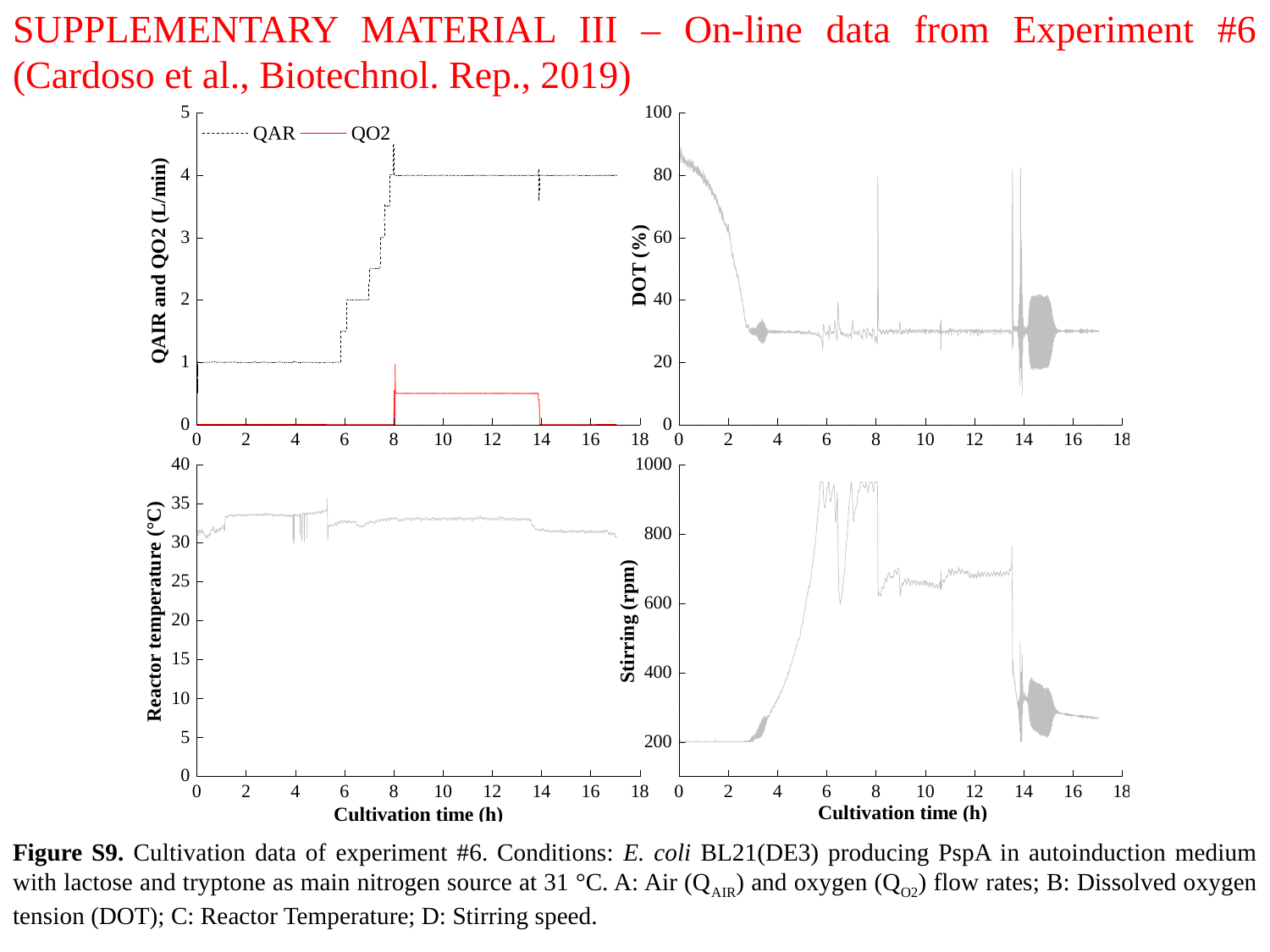

SUPPLEMENTARY MATERIAL III – On-line data from Experiment #6 (Cardoso et al., Biotechnol. Rep., 2019)
Figure S9. Cultivation data of experiment #6. Conditions: E. coli BL21(DE3) producing PspA in autoinduction medium with lactose and tryptone as main nitrogen source at 31 °C. A: Air (QAIR) and oxygen (QO2) flow rates; B: Dissolved oxygen tension (DOT); C: Reactor Temperature; D: Stirring speed.

## Slide 11
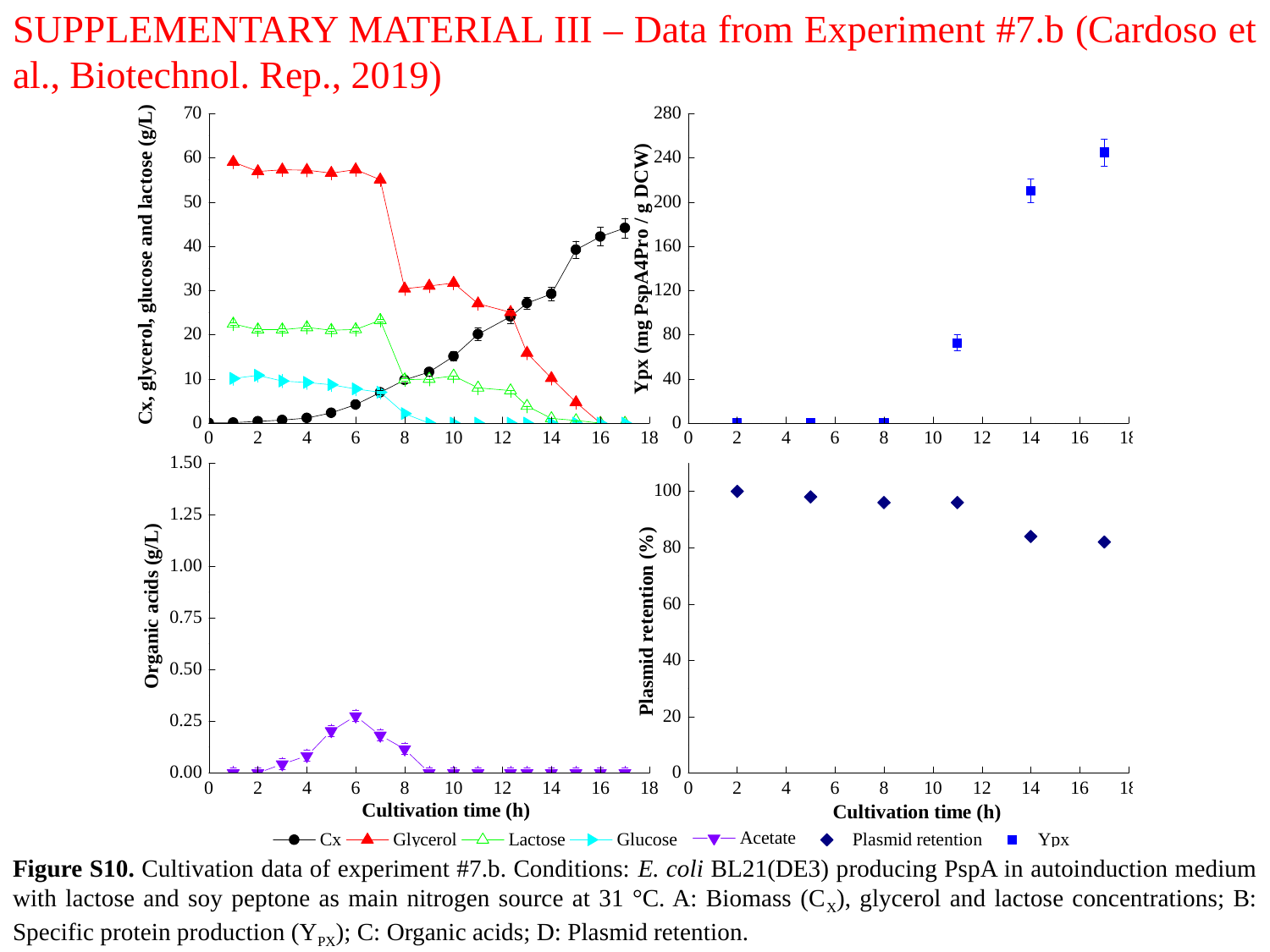

SUPPLEMENTARY MATERIAL III – Data from Experiment #7.b (Cardoso et al., Biotechnol. Rep., 2019)
Figure S10. Cultivation data of experiment #7.b. Conditions: E. coli BL21(DE3) producing PspA in autoinduction medium with lactose and soy peptone as main nitrogen source at 31 °C. A: Biomass (CX), glycerol and lactose concentrations; B: Specific protein production (YPX); C: Organic acids; D: Plasmid retention.

## Slide 12
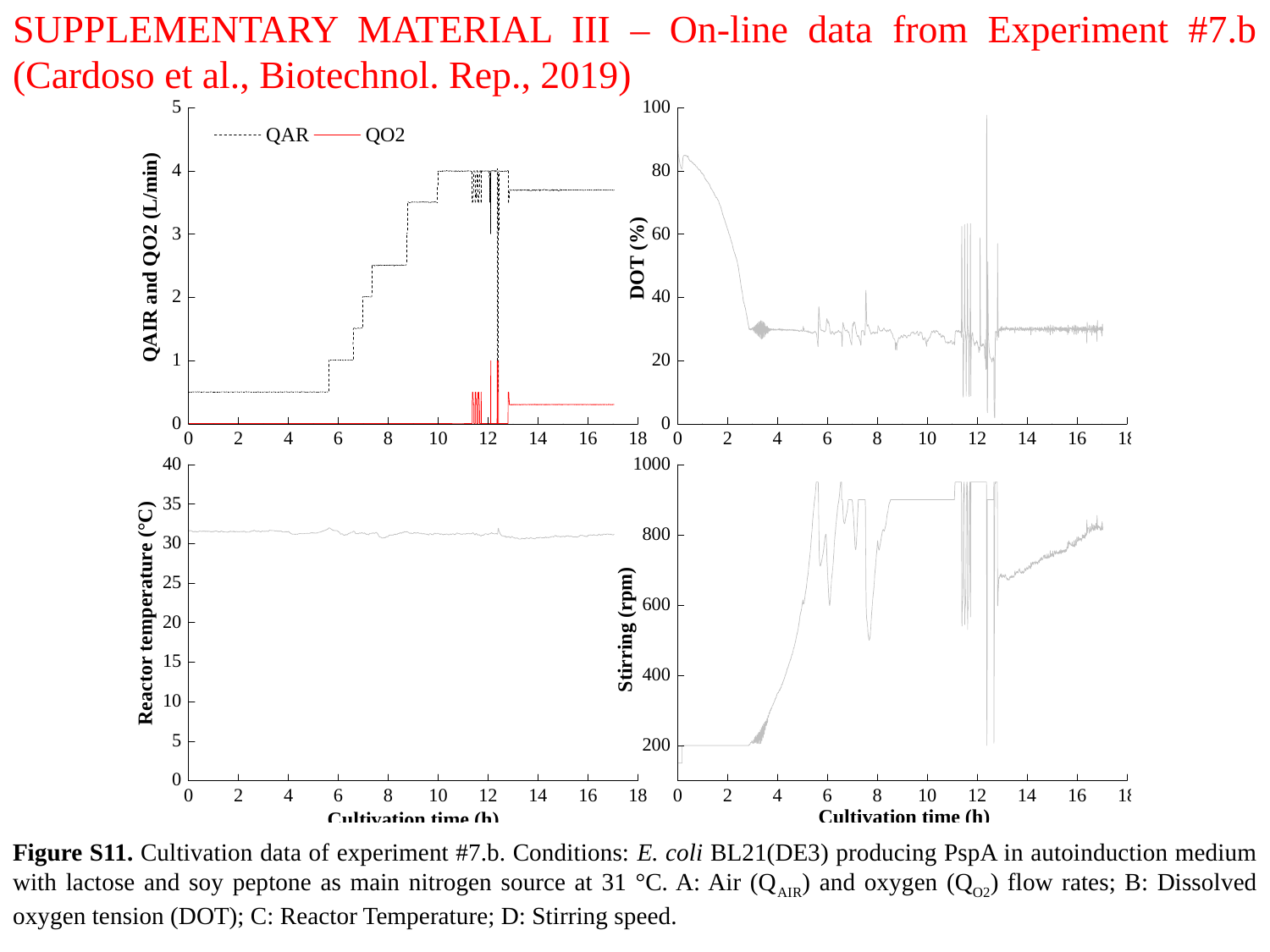

SUPPLEMENTARY MATERIAL III – On-line data from Experiment #7.b (Cardoso et al., Biotechnol. Rep., 2019)
Figure S11. Cultivation data of experiment #7.b. Conditions: E. coli BL21(DE3) producing PspA in autoinduction medium with lactose and soy peptone as main nitrogen source at 31 °C. A: Air (QAIR) and oxygen (QO2) flow rates; B: Dissolved oxygen tension (DOT); C: Reactor Temperature; D: Stirring speed.

## Slide 13
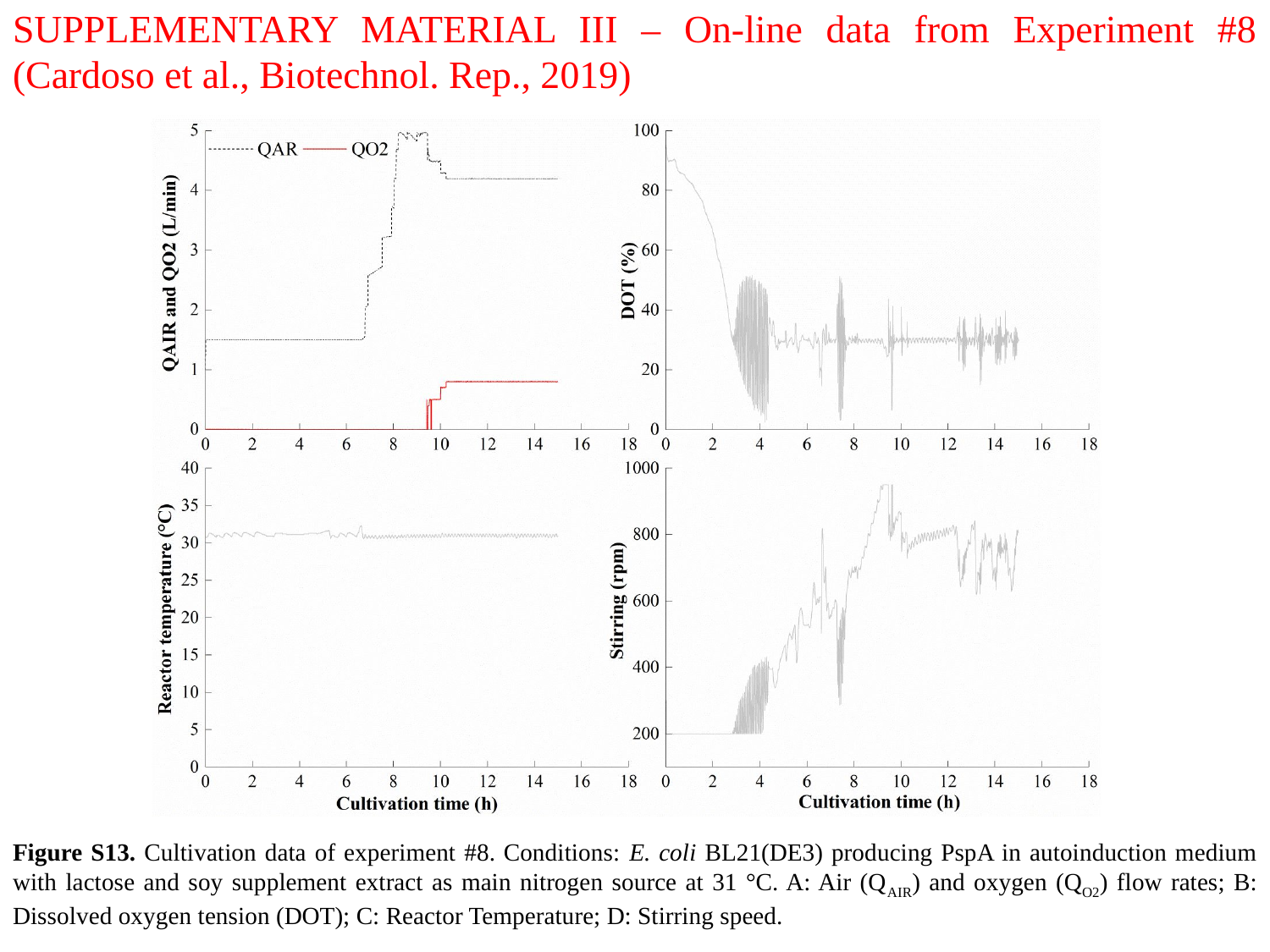

SUPPLEMENTARY MATERIAL III – On-line data from Experiment #8 (Cardoso et al., Biotechnol. Rep., 2019)
Figure S13. Cultivation data of experiment #8. Conditions: E. coli BL21(DE3) producing PspA in autoinduction medium with lactose and soy supplement extract as main nitrogen source at 31 °C. A: Air (QAIR) and oxygen (QO2) flow rates; B: Dissolved oxygen tension (DOT); C: Reactor Temperature; D: Stirring speed.

## Slide 14
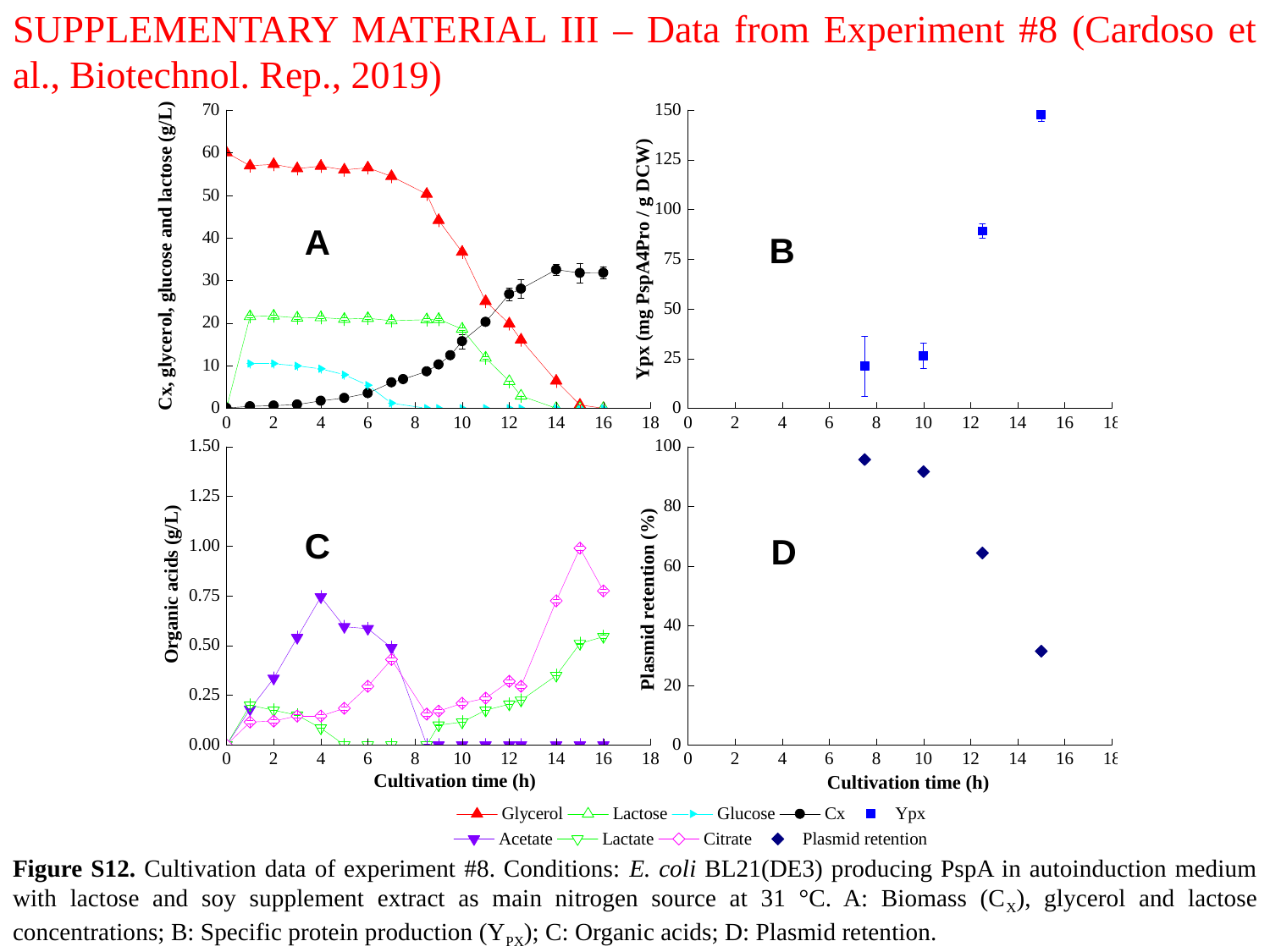

SUPPLEMENTARY MATERIAL III – Data from Experiment #8 (Cardoso et al., Biotechnol. Rep., 2019)
Figure S12. Cultivation data of experiment #8. Conditions: E. coli BL21(DE3) producing PspA in autoinduction medium with lactose and soy supplement extract as main nitrogen source at 31 °C. A: Biomass (CX), glycerol and lactose concentrations; B: Specific protein production (YPX); C: Organic acids; D: Plasmid retention.

## Slide 15
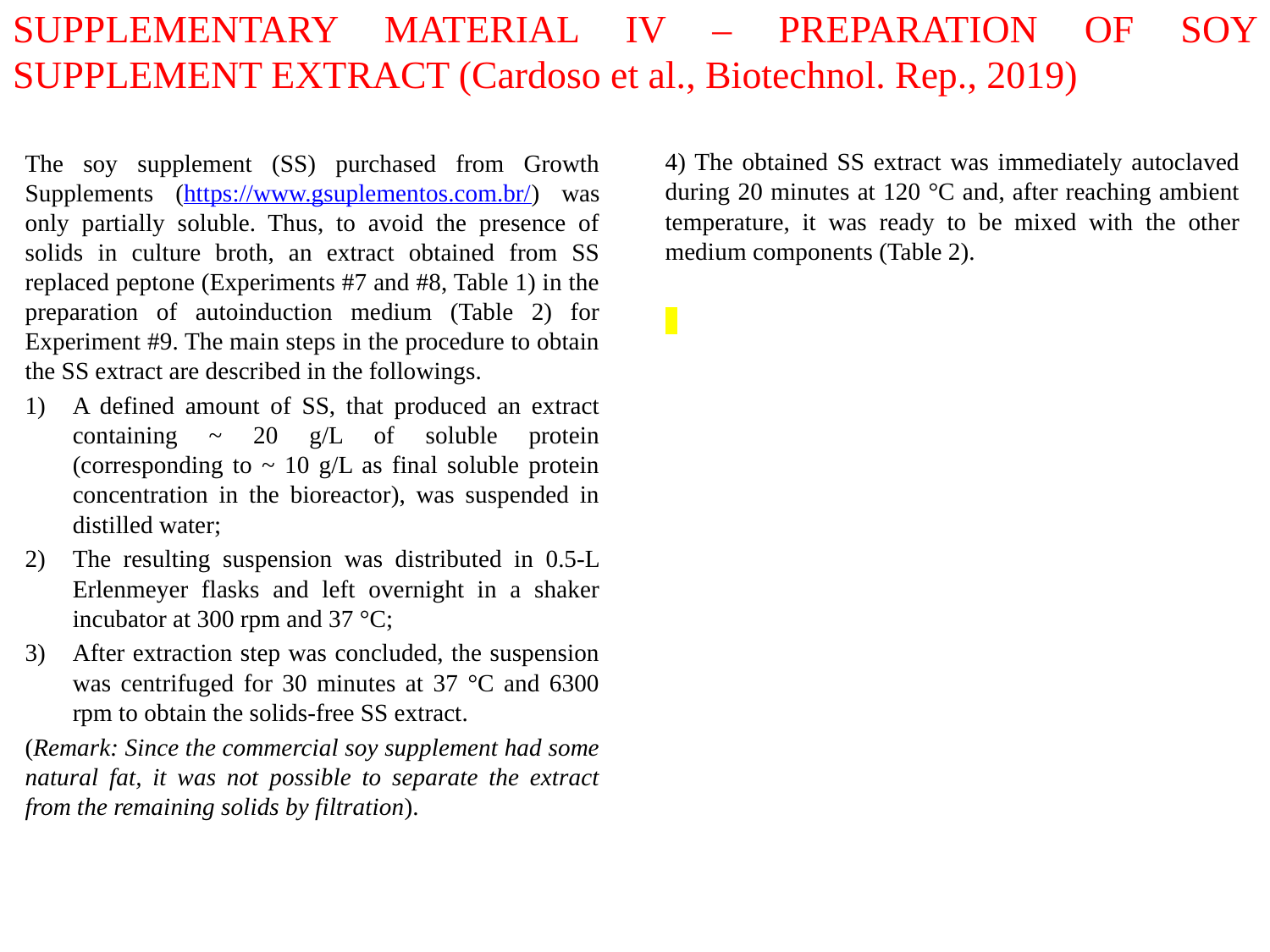

SUPPLEMENTARY MATERIAL IV – PREPARATION OF SOY SUPPLEMENT EXTRACT (Cardoso et al., Biotechnol. Rep., 2019)
4) The obtained SS extract was immediately autoclaved during 20 minutes at 120 °C and, after reaching ambient temperature, it was ready to be mixed with the other medium components (Table 2).
The soy supplement (SS) purchased from Growth Supplements (https://www.gsuplementos.com.br/) was only partially soluble. Thus, to avoid the presence of solids in culture broth, an extract obtained from SS replaced peptone (Experiments #7 and #8, Table 1) in the preparation of autoinduction medium (Table 2) for Experiment #9. The main steps in the procedure to obtain the SS extract are described in the followings.
A defined amount of SS, that produced an extract containing ~ 20 g/L of soluble protein (corresponding to ~ 10 g/L as final soluble protein concentration in the bioreactor), was suspended in distilled water;
The resulting suspension was distributed in 0.5-L Erlenmeyer flasks and left overnight in a shaker incubator at 300 rpm and 37 °C;
After extraction step was concluded, the suspension was centrifuged for 30 minutes at 37 °C and 6300 rpm to obtain the solids-free SS extract.
(Remark: Since the commercial soy supplement had some natural fat, it was not possible to separate the extract from the remaining solids by filtration).
